# Gd_2_O_3_ Doped with Yb^3+^/Er^3+^ for Boosted Downshifting Pathway in NIR-IIb Region and Exploring the Dynamics of MRI/NIR-II Imaging in the Nanophosphor

**DOI:** 10.1021/acsami.5c14860

**Published:** 2025-10-22

**Authors:** Aishwarya Satpathy, Tzu-Hsuan Liu, Ting-Yi Su, Shiqi Yu, Wei Zhang, Datao Tu, Agata Lazarowska, Natalia Majewska, Grzegorz Leniec, Ewa Mijowska, Xueyuan Chen, Sebastian Mahlik, Ming-Hsien Chan, Ru-Shi Liu

**Affiliations:** a Department of Chemistry, National Taiwan University, Taipei 106, Taiwan; b State Key Laboratory of Structural Chemistry, and Fujian Key Laboratory of Nanomaterials, Fujian Institute of Research on the Structure of Matter, Chinese Academy of Sciences, Fuzhou, Fujian 350002, China; c Institute of Experimental Physics, Faculty of Mathematics, Physics and Informatics, University of Gdansk, Wita Stwosza 57, 80-308 Gdansk, Poland; d Faculty of Chemistry, Adam Mickiewicz University, Uniwersytetu Poznańskiego 8, 61-614 Poznań, Poland; e Department of Nanomaterials Physicochemistry, Faculty of Chemical Technology and Engineering, West Pomeranian University of Technology, Piastow Ave. 45, 70-311 Szczecin, Poland; f Center for Advanced Materials and Manufacturing Process Engineering, West Pomeranian University of Technology, 70-310 Szczecin, Poland; g Department of Biomedical Imaging and Radiological Sciences, National Yang Ming Chiao Tung University, Taipei 112, Taiwan

**Keywords:** lanthanides, downshifting, NIR-IIb, magnetic resonance imaging, liver imaging

## Abstract

Lanthanide-ion-activated nanoparticles stimulated by 808 or 980 nm lasers present promising applications in biological imaging. This contribution reveals their physicochemical properties and explores their potential as near-infrared-II (NIR-II) fluorescent agents for bioimaging. Specifically, the NIR-IIb window (1500–1700 nm) has the advantages of low scattering and less autofluorescence from the tissues, which makes this region suitable for imaging with greater clarity. Lanthanides offer diverse emission possibilities due to their rich energy levels, which make them highly effective nanoprobes. This study focuses on gadolinium oxide (Gd_2_O_3_) as the host material due to its facile fabrication and low toxicity. The Gd_2_O_3_ system is doped with Yb^3+^ and Er^3+^ ions and achieves a high quantum efficiency of 22.8% in the NIR-IIx and NIR-IIb windows. Moreover, the superior penetrability of the NIR-IIb window is unveiled by the penetration depth testing and *in vivo* imaging studies. Additionally, Gd^3+^ ions exhibit magnetic properties, which support their application in magnetic resonance imaging (MRI). This work reveals the high brightness and high energy transfer efficiency of the Yb^3+^–Er^3+^ system and explores the feasibility of Gd_2_O_3_ nanoparticles in MRI. Therefore, we believe that this work provides a superior and biocompatible candidate for understanding the dynamics of MRI/NIR-II imaging of the nanophosphor for clinical applications.

## Graphical abstract

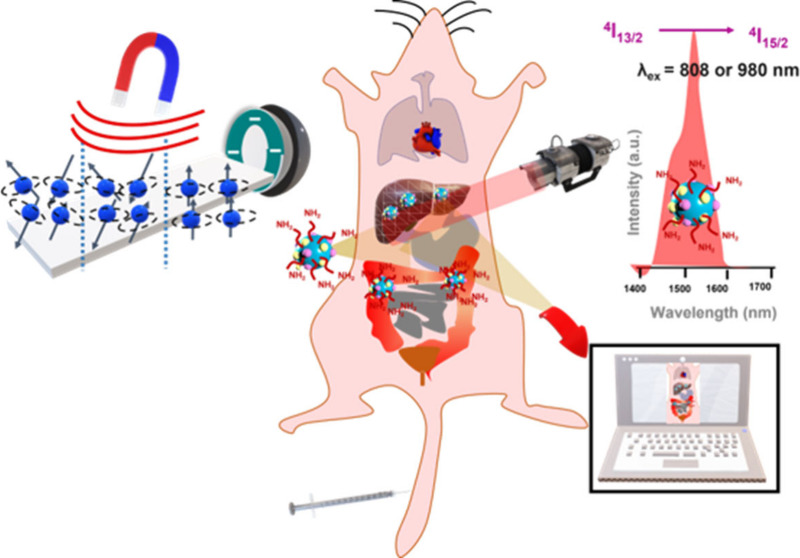

## Introduction

An extremely sensitive and satisfactory fluorescent nanoprobe is a prerequisite for the early diagnosis and treatment of diseases.^1–3^ Reducing the background signals of normal tissue from the target organs enhances the credibility of the exogenous nanomaterials.^4^ Combining multiple imaging modalities provides a powerful approach to address the limitations of individual techniques and bridges the gap between preoperative imaging and intraoperative conditions.^5^ This integration enhances diagnostic precision, offers real-time guidance during surgical procedures, and ensures more accurate and effective clinical outcomes in various medical applications.^6^ Numerous developments of multimodal nanoparticles have caught the attention of the biomedical field due to their ability to diagnose diseases with utmost clarity and precision.^7–9^ The strengths of one imaging technique can be complemented by those of another, which minimizes both drawbacks. The most widely used nanostructured multimodal imaging probes integrate magnetic resonance imaging (MRI) with near-infrared-II (NIR-II) fluorescence imaging, providing enhanced diagnostic capabilities and imaging performance for biomedical applications.^10^ Rare-earth-doped nanoparticles featuring lanthanide ions within an inorganic crystalline host matrix, such as Gd_2_O_3_, CaF_2_, NaCeF_4_, or NaYF_4_, have garnered significant interest as NIR-II fluorophores.^9,11–13^ Their appeal lies in features such as extended lifetimes, minimal Stokes shifts, narrow multipeak emissions, and extraordinary photostability.^14^ Compared with the most common crystalline fluoride host matrices such as CaF_2_ ^15^ and NaYF_4_,^16^ Gd_2_O_3_ as a host material possesses low water solubility and stability at high temperatures.^17^ Gd_2_O_3_ is also an environmentally friendly material that has no extra fluorescence peaks from the visible to the NIR region.^18^ Notably, the suppression of tissue autofluorescence contributes to remarkably enhanced signal-to-noise ratios. NIR-II optical probes with high quantum efficiency provide high spatial resolution due to their satisfactory penetration depth, decreased autofluorescence, and increased sensitivity.^19^ MRI provides more precise relative positioning details compared to other imaging techniques and enhances the biological insights obtained from them.^20,21^ Fabricating nanoparticles with two or more systems sometimes leads to complex synthesis techniques and quenching effects. To address this problem, Gd-based nanosystems provide a favorable single-phase multifaceted probe that confines the optical and magnetic properties. Gd_2_O_3_ is an effective MRI contrast agent due to its magnetic properties and is an excellent host for rare-earth luminescent materials because its low-phonon energy reduces nonradiative losses, thereby enhancing luminescence efficiency.^22,23^ Doan Thi Kim Dung et al.^24^ elaborated on the utility of Gd_2_O_3_ nanophosphors as a host for doping with different lanthanide ions for upconversion and downconversion imaging properties. Guo et al.^25^ showed the work on the upconversion pathway of the Gd_2_O_3_ system with varying ions of lanthanide (Ln^3+^ = Yb, Er, and Tm). Later, Yadaw et al.^26^ demonstrated the upconversion pathway of Gd_2_O_3_:Yb^3+^,Er^3+^ system for magnetic resonance application. In 2019, Liu et al.^27^ showed the energy transfer phenomenon of Yb^3+^ and Er^3+^ for the Gd_2_O_3_ host, while the emission was from 750–1200 nm, instead of the NIR-IIb region. These works confirmed the ability of Gd_2_O_3_ to serve as a host for a multimodality probe. However, using Gd_2_O_3_ as a host and exploring the Yb^3+^/Er^3+^ energy transfer pathway to enhance the NIR-IIb emission are less addressed. These works confirmed the ability of the Gd_2_O_3_ host as a multimodality probe.

In this study, the energy transfer pathway from the Yb^3+^ to Er^3+^ is explored using in-depth optical studies, which increased the internal quantum efficiency (IQE) of the Gd_2_O_3_:Yb^3+^,Er^3+^ system to greater than 40% within the NIR region and more than 22% in the window of the NIR-IIx (1400–1500 nm)^28^ and NIR-IIb (1500–1700 nm) regions, with an expected higher signal-to-noise ratio and a more extended penetration depth. The coating of (3-aminopropyl)­triethoxysilane (APTES) improved the dispersibility of the nanoparticles. The nanosized material contains the MRI active property of Gd^3+^ and the NIR-IIb emission of Yb^3+^–Er^3+^ ions. Our system showed a downshifting pathway because of the absorption of a photon with higher energy and leading to the emission of a photon with lower energy, which is different from the downconversion (quantum cutting) pathway, as it leads to the emission of two or more photons with lower energy after excitation with a higher energy photon, and is a little challenging to implement.^29,30^ The coating with APTES enabled the nanoparticle to be injected into the mouse body via the intravenous (iv) route to observe the organs using MRI/NIR-II imaging for precise, reliable, and fast diagnosis of diseases. We believe that our strategy can also be utilized for other clinical applications.

## Results and Discussion

### Nanoparticle Characterization

The citric acid sol–gel process to synthesize the Gd_2_O_3_:*x*Yb^3+^,*y*Er^3+^ (*x* = 0.025, and *y* = 0.01) system followed by sintering at a high temperature of 800 °C is represented in Figure 1a, where after the synthesis of Gd_2_O_3_:0.025Yb^3+^,0.01Er^3+^ (GOYE) system, APTES coating was performed to improve the dispersibility of the GOYE system in aqueous solutions for use as a bioimaging agent. The XRD patterns for the different concentrations of Yb^3+^ = 1%, 2.5%, 5%, 10%, and 15% showed a pure phase that matched the JCPDS pattern of JCPDS 12-0797 (Figure 1b). No extra impurities were seen in the XRD patterns. Figure 1b also refers to the XRD patterns of a fixed concentration of Yb^3+^ = 2.5% and varying concentrations of Er^3+^ = 0.1%, 0.5%, 1%, 1.5%, and 2%. Gd_2_O_3_ acquired a cubic structure with a space group of *Ia* 3. Two differing Gd^3+^ sites were observed. The first site had Gd^3+^ ions bonded to the six O^2–^ atoms and shared the edges with the octahedral [GdO_6_]. However, the octahedral [GdO_6_] site was distorted in the second site. The oxygen atoms were located on the Wyckoff site 24*d* (Figure 1c). The lattice parameters of Gd_2_O_3_ had *a* = *b* = *c* values at 10.79 Å and a unit cell volume of 1256.21 Å^3^. The in-depth high-resolution transmission electron microscopy (HRTEM) analysis with average particle distribution is exhibited in the Supporting Information. Figure S1a corresponds to the selected area diffraction (SAED) pattern of the GOYE lanthanide system because this sample had the highest NIR-II emission intensity (Gd_2_O_3_:0.025Yb^3+^,0.01Er^3+^). The SAED patterns conformed to a pattern confirming the (211), (231), and (200) planes of the Gd_2_O_3_ crystal system. Figure S1b shows the dark field lattice fringes, which were analyzed using the fast Fourier transform (FFT) and validated the crystallinity of the Gd_2_O_3_ crystal. The EDS analysis corroborated the Gd, O, Yb, and Er elements, as presented in Figure S1c. The TEM analysis confirmed that the average size of the GOYE nanoparticles was 28.72 ± 2.98 nm, which was calculated using ImageJ software (Figure S1d). Figure 1d corroborates the uniform coating of APTES around the GOYE nanoparticle with a uniform coating thickness of 3–5 nm. Moreover, the GOYE nanoparticles showed a homogeneous circular structure. Gd_2_O_3_:0.025Yb^3+^,0.01Er^3+^ (referred to as GOYE in the subsequent sections) samples were used for the other characterizations because they possess the highest NIR-II emission intensity among the samples with different dopant concentrations.

**Figure 1:**
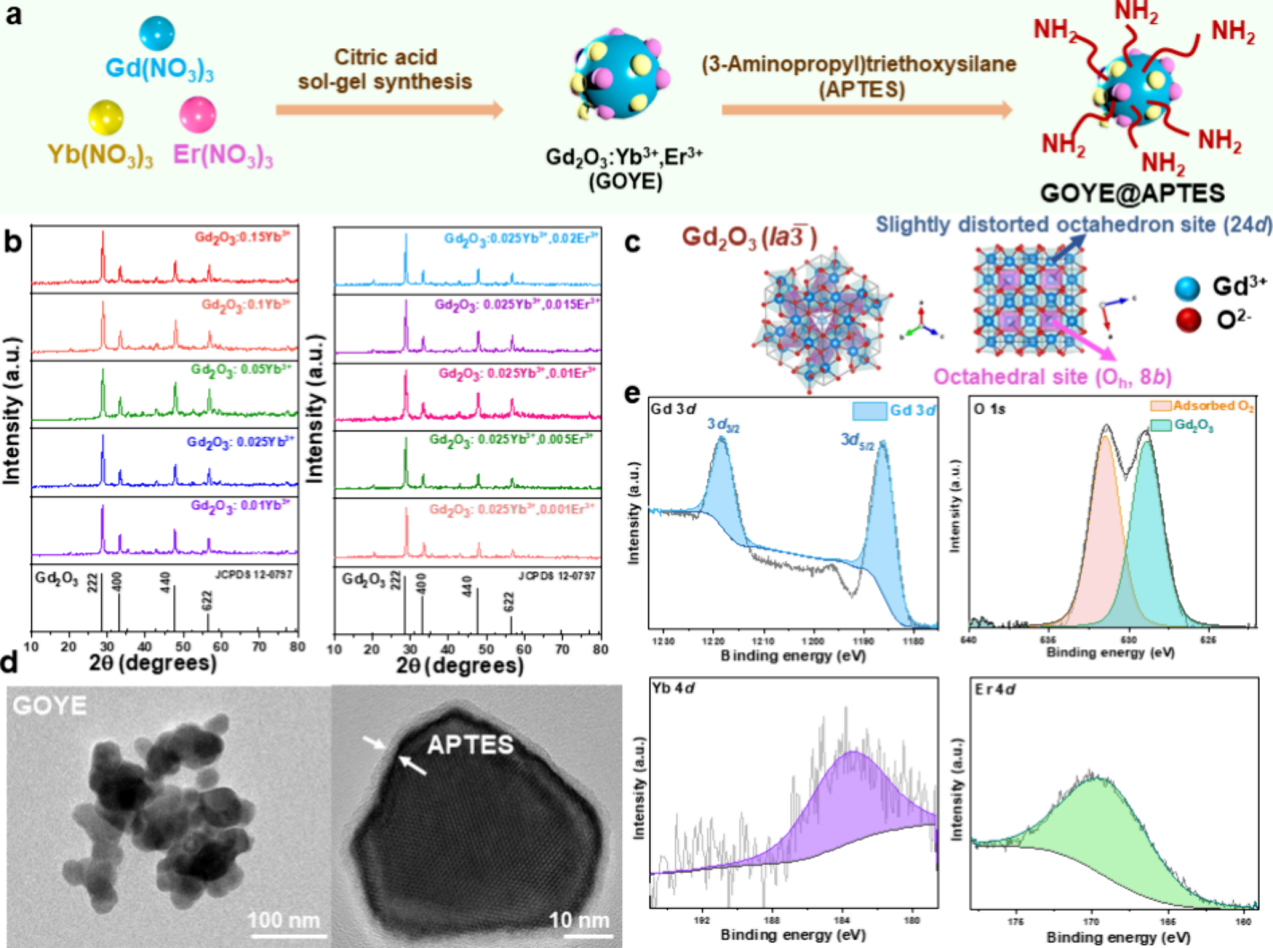
Gd_2_O_3_:*x*Yb^3+^,*y*Er^3+^ nanoparticle characterization: (a) scheme representing the synthesis and coating of the Gd_2_O_3_:*x*Yb^3+^,*y*Er^3+^ nanoparticle with APTES; (b) XRD patterns of the Gd_2_O_3_:*x*Yb^3+^,*y*Er^3+^ nanoparticle, with varying concentrations of Yb and Er; (c) crystal structure of the Gd_2_O_3_ unit cell drawn using VESTA software; (d) HRTEM images of Gd_2_O_3_:0.025Yb^3+^,0.01Er^3+^ (GOYE) and GOYE@APTES nanoparticles; (e) GOYE lanthanide system XPS fitting for Gd 3d and O peaks, Yb 4d, and Er 4d peaks.

X-ray photoelectron spectroscopy (XPS) analysis using a Gaussian–Lorentzian peak shape and linear background correction confirmed the chemical composition of the sample and bonding structure. The results verified the presence of Er^3+^ and Yb^3+^ ions within the material. A binding energy peak at 168 eV was attributed to the 4d valence state of Er^3+^, which confirmed its incorporation into the sample. Similarly, a peak at 183 eV corresponded to the 4d valence state of Yb^3+^, which further substantiated the inclusion of Yb^3+^. The formation of Gd_2_O_3_ was also confirmed by the presence of characteristic peaks for Gd 3d_5/2_ and Gd 3d_3/2_ at binding energies of 1186 and 1218 eV, respectively. These peaks indicated that gadolinium was in its expected oxidation state and formed a part of the Gd_2_O_3_ structure. Further evidence of successful bonding was provided by the O 1s spectrum, which showed a primary peak at 528 eV, consistent with the Gd–O bonding characteristic of Gd_2_O_3_. A secondary peak at 531 eV was also identified as adsorbed O_2_, which suggested surface interactions within the sample. In Figure 1e, the XPS analysis results validate the successful incorporation of Er^3+^ and Yb^3+^. The findings confirmed the chemical composition and oxidation state of the existing ions. The analysis highlighted the adequate bonding of gadolinium and oxygen to form Gd_2_O_3_ while demonstrating the successful inclusion of the +3 oxidation state of erbium and ytterbium ions. The APTES coating on the GOYE nanoparticle was confirmed by Fourier transform infrared (FTIR) measurements (Figure S2). In the uncoated Gd_2_O_3_, a peak observed around 841 cm^–1^ corresponded to the Gd–O vibrational mode, which confirmed the presence of Gd_2_O_3_.^31^ By contrast, the APTES-coated Gd_2_O_3_ showed a new, low-intensity peak of around 1637 cm^–1^,^32^ which can be attributed to the −NH_2_ deformation introduced by the APTES layer.^33,34^ This additional peak proved the successful APTES coating on the Gd_2_O_3_ nanoparticle system and demonstrated that the surface was effectively modified with the aminosilane compound. This detailed characterization underscored the synthesis’s precision and effectiveness and ensured the material’s intended composition and structure were achieved. Through this comprehensive analysis, this study established the chemical and structural framework necessary for the further application and development of the material.

### Optical Property Analysis and Structural Investigation

The downshifting process of Gd_2_O_3_:*x*Yb^3+^,*y*Er^3+^ nanoparticles can be explained by the multiphonon relaxation process (denoted as n1 in Figure 2a) that takes place from the ^4^I_9/2_ and ^4^I_11/2_ to the ^4^I_13/2_ emitting state. The excitation by the 980 nm laser led to the electron excitation from the ^4^I_15/2_ ground state to the ^4^I_11/2_ excited state of the Er^3+^ ions, and the electron excitation from the ^2^F_7/2_ ground state to ^2^F_5/2_ excited state of the Yb^3+^ ion. Due to the close energy values of the ^2^F_5/2_ states of Yb^3+^ and the ^4^I_11/2_ states of Er^3+^, the energy may be transferred from Yb^3+^ to Er^3+^ ions. When Gd_2_O_3_:*x*Yb^3+^,*y*Er^3+^ is illuminated with 980 nm light, Yb^3+^ ions are predominantly absorbing light, following the energy transfer to the ^4^I_11/2_ state of Er^3+^, which then by a multiphonon relaxation process (n1), leads to the population of the ^4^I_13/2_ state and subsequent producing emission with a maximum at 1540 nm during radiative relaxation process to the ground state of Er^3+^ ions. However, at 808 nm, excitation directly populates the ^4^I_9/2_ level of Er^3+^, which subsequently relaxes to intermediate states (such as ^4^I_11/2_ or ^4^I_13/2_) and participates in sequential energy transfer or excited-state absorption to generate visible or NIR emission. This process is governed by the relatively weak absorption cross-section of Er^3+^ ions at 808 nm, meaning that efficient population of the emitting states depends strongly on direct photon absorption by Er^3+^. Thus, the mechanistic difference lies in whether Er^3+^ ions are excited directly at 808 nm or indirectly through Yb^3+^ sensitization at 980 nm, with the latter generally providing higher excitation efficiency and stronger luminescence.^35^ The PLE spectra comparison for 808 and 980 nm excitation after λ_em_ = 1540 nm for GOYE, Y_2_O_3_:0.025Yb^3+^,0.01Er^3+^ (YOYE), and NaYF_4_:0.18Yb^3+^,0.01Er^3+^ (NaYFYE) nanophosphors is shown in Figure S3. Figure S4a presents the photoluminescence (PL) spectra in the NIR spectral region (1500–1600 nm) of the Gd_2_O_3_:*x*Yb^3+^,*y*Er^3+^ with *x* = 1%, 2.5%, 5%, 10%, 15% and *y* = 1%, after 980 nm excitation. Gd_2_O_3_:0.025Yb^3+^,0.01Er^3+^ lanthanide system had the highest emission intensity compared with the other concentrations of Yb^3+^. Figure S4b illustrates the PL spectra of Gd_2_O_3_:*x*Yb^3+^,*y*Er^3+^, where *x* is 2.5% and y varies between 0.1%, 0.5%, 1%, 1.5%, and 2%. Across this range of lanthanide-doped samples, a consistent pattern of NIR emission was observed. Among the compositions analyzed, Gd_2_O_3_:0.025Yb^3+^ and 0.01Er^3+^ demonstrated the highest emission intensity, which indicated superior luminescence performance. This outcome suggests that fine-tuning the doping concentrations of Yb^3+^ and Er^3+^ significantly influences the optical properties of Gd_2_O_3_, and this specific combination yielded the most efficient luminescence. Figure S5a,b corresponds to the lanthanide system’s IQE measurements, which matched the PL spectra results. The highest quantum yield value of 41.1% was obtained for the Gd_2_O_3_:0.025Yb^3+^,0.01Er^3+^ material in the total (NIR-I and NIR-II) region after excitation with a 980 nm laser. The NIR-I and NIR-II regions had quantum yields of 18.3% and 22.8%, respectively. Figure 2b presents the room-temperature (RT) photoluminescence excitation (PLE) spectra of Gd_2_O_3_:0.025Yb^3+^, Gd_2_O_3_:0.01Er^3+^, and Gd_2_O_3_:0.025Yb^3+^,0.01Er^3+^, monitored at emission wavelengths of 1070, 1540, and 1540 nm, respectively. In the PLE spectrum of Gd_2_O_3_:0.025Yb^3+^ (orange curve), observed at 1070 nm, characteristic lines for Yb^3+^ ions appeared in the 850–1000 nm range, which corresponded to the ^2^F_7/2_ → ^2^F_5/2_ transition of Yb^3+^ ions. The PLE spectrum of Gd_2_O_3_:0.01Er^3+^ (green curve) revealed several distinct peaks at 382, 525, 658, 801, and 980 nm that corresponded to Er^3+^ transitions ^4^I_15/2_ → ^4^G_11/2_, ^2^H_11/2_, ^4^F_9/2_, ^4^I_9/2_, and ^4^I_11/2_, respectively. The intensity of the 980 nm excitation line of Er^3+^ is very low. At the same time, the intensity of 980 nm excitation lines of Yb^3+^ is high, which suggests that the participation of Yb^3+^ ions could increase the absorption of 980 nm light compared with the system of Er^3+^ ions alone. For the co-doped sample Gd_2_O_3_:0.025Yb^3+^,0.01Er^3+^ (purple curve), the PLE spectrum observed at 1540 nm, which corresponds to the Er^3+^ ^4^I_13/2_ → ^4^I_15/2_ transition, is composed of spectral features from the samples doped individually with Er^3+^ and Yb^3+^ ions. Notably, the presence of Yb^3+^ peaks (^2^F_7/2_ → ^2^F_5/2_) within the 900–1000 nm range in the PLE spectrum (monitoring Er^3+^ luminescence) of co-doped samples indicates the energy transfer process between the Yb^3+^ and Er^3+^ ions. The energy transfer process enhances the emission intensity of Er^3+^ ions with the maximum at 1540 nm and maximizes the luminescence efficiency in the NIR spectral region. Figure 2c displays the PL spectra of Gd_2_O_3_:0.025Yb^3+^ under excitations at 915 nm, as well as Gd_2_O_3_:0.01Er^3+^ and Gd_2_O_3_:0.025Yb^3+^,0.01Er^3+^ under excitations at 980 nm. When excited at 915 nm, the Gd_2_O_3_:0.025Yb^3+^ sample (orange curve) exhibited several NIR emission lines in the 950–1100 nm range, which corresponded to transitions between the ^2^F_5/2_ and ^2^F_7/2_ states of Yb^3+^. Note that the Yb^3+^ energy levels of the 4f^13^ configuration and the Er^3+^ energy levels of the 4f^11^ configuration consist of multiple closely spaced Stark sublevels, arising from splitting these levels by the crystal field effect induced by the host lattice. For the Gd_2_O_3_:0.01Er^3+^ sample excited at 980 nm, multiple NIR emission peaks were observed (green curve), particularly around 1000 nm and in the 1500–1600 nm spectral range with the maximum at 1540 nm. These lines correspond to transitions between sublevels within Er^3+^ manifolds ^4^I_11/2_ → ^4^I_15/2_ and ^4^I_13/2_ → ^4^I_15/2_, respectively. The PL spectrum of the codoped sample, Gd_2_O_3_:0.025Yb^3+^,0.01Er^3+^ (purple curve), under 980 nm excitation showed overlapping emissions from Yb^3+^ (^2^F_5/2_ → ^2^F_7/2_) and Er^3+^ (^4^I_11/2_ →^4^I_15/2_) in the 1000–1100 nm range and the dominant Er^3+^ emission with a maximum at 1540 nm, attributed to the ^4^I_13/2_ → ^4^I_15/2_ transition. As previously stated, enhancement of the Er^3+^ emission at 1540 nm for Gd_2_O_3_:0.025Yb^3+^,0.01Er^3+^ is a consequence of energy transfer from Yb^3+^ to Er^3+^ ions following a nonradiative transition (n1) from the ^4^I_11/2_ and ^4^I_9/2_ states to ^4^I_13/2_ in Er^3+^ (Figure 2a). Figure S6 shows part of the PL spectra in the 920 to 1100 nm range, excited at 915 nm of Gd_2–*x*_O_3_:*x*Yb^3+^ samples with different Yb^3+^ concentrations. Also, the two horizontal axes for Figure 2b and Figure 2c are due to the relation between the wavelength and wavenumber, which is related by eq 1:

1 v=1/λ

**Figure 2:**
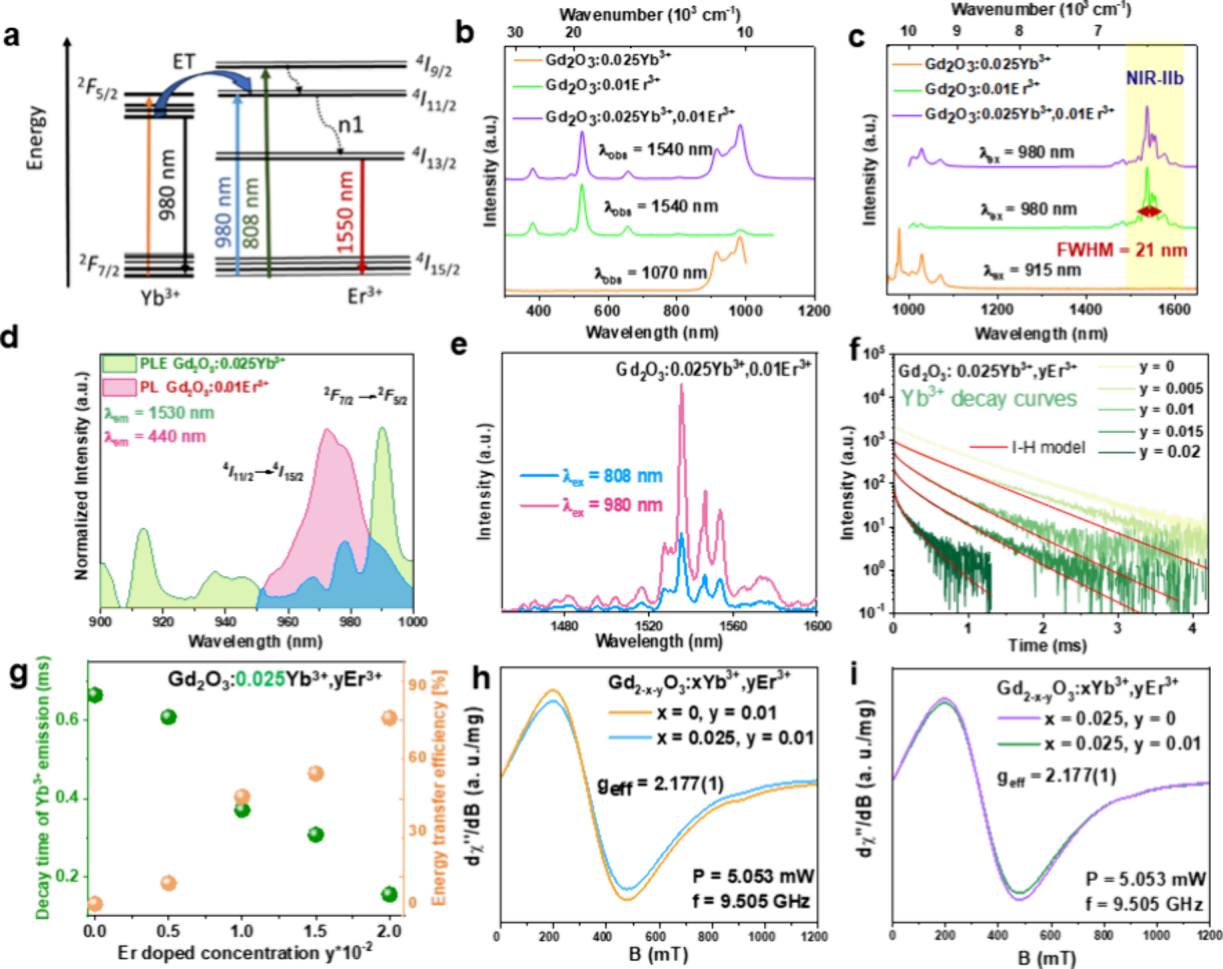
(a) Schematic illustration of energy transfer mechanism from Yb^3+^ ions to Er^3+^ ions. (b) RT PLE spectra of Gd_2_O_3_:0.025Yb^3+^ were observed at 1070 nm, and Gd_2_O_3_:0.01Er^3+^ and Gd_2_O_3_:0.025Yb^3+^,0.01Er^3+^ were observed at 1540 nm. (c) RT PL spectra of Gd_2_O_3_:0.025Yb^3+^, Gd_2_O_3_:0.01Er^3+^, and Gd_2_O_3_:0.025Yb^3+^,0.01Er^3+^ excited at 915 and 980 nm, respectively. (d) Spectral overlap between PLE of Gd_2_O_3_:0.01Er^3+^ at λ_em_ = 1530 nm and PL of Gd_2_O_3_:0.025Yb^3+^ at λ_ex_ = 980 nm. (e) PL plot of Gd_2_O_3_:0.025Yb^3+^,0.01Er^3+^ sample after excitation with the laser of λ_ex_ = 980 nm and λ_ex_ = 800 nm. (f) PL decay profiles of Yb^3+^ emission in Gd_2_O_3_:0.025Yb^3+^,*y*Er^3+^ (*y* = 0, 0.005, 0.01, 0.015, 0.02) excited at 980 nm. Red curves represent the fits using the I–H model. (g) Decay times of Yb^3+^ were obtained by eq 2, and energy transfer efficiency increased as the concentration of Er^3+^ increased. (h) EPR spectra for the Gd_2_O_3_ compound for fixed Er^3+^ ions concentrations. (i) EPR spectra for the Gd_2_O_3_ compound for fixed Yb^3+^ ions concentrations.

Figure 2d presents the spectral overlap between the excitation spectra of Gd_2_O_3_:0.01Er^3+^ at λ_em_ = 1540 nm and the emission spectra of Gd_2_O_3_:0.025Yb^3+^ at λ_ex_ = 980 nm. In Figure 2e, the emission at 1540 nm after excitation with 808 nm had a lower intensity (blue line) than after 980 nm excitation (pink line) by almost three times. This shows the importance of 980 nm laser excitation, which enhanced the NIR-II signal and the quality of the images. Figure 2f shows the decay curves of Yb^3+^ PL in Gd_2_O_3_:0.025Yb^3+^,*y*Er^3+^ with varying concentrations *y* of Er^3+^ ions. As the concentration of Er^3+^ ions increased, the decay rate of the Yb^3+^ emission became faster. The decay profiles were multiexponential, so the average decay time was calculated using eq 2.

2 $$\tau_{\mathrm{av}}=\frac{\int tI\left(t\right)dt}{\int I\left(t\right)dt}$$

where *I*(*t*) is the emission intensity at time *t*.

Figure 2g illustrates the relationship between Er^3+^ doping concentration and the decay time of Yb^3+^ emission (observation at 1000 nm), and the energy transfer efficiency η between the Yb^3+^ and Er^3+^ ions in Gd_2_O_3_. When Er^3+^ was present, Yb^3+^ transferred energy nonradiatively to Er^3+^, which accelerated the decay of the Yb^3+^ emission. The shorter decay time of Yb^3+^ in the presence of Er^3+^ indicated a more efficient energy transfer. The closer the value of η to 1 (or 100%), the more effective is the energy transfer. Energy transfer efficiency was defined as η = 1 – τ­(Yb)/τ­(Yb­(Er)), where τ­(Yb) is the intrinsic decay time of Yb^3+^ emission in the absence of Er^3+^, and τ­(Yb­(Er)) is the decay time of Yb^3+^ emission in the presence of Er^3+^ ions. The decay time of Yb^3+^ emission (left *y*-axis) decreased with increasing Er^3+^ concentration, while the energy transfer efficiency (right *y*-axis) increased correspondingly. In samples with no Er^3+^ doping (*y* = 0), the Yb^3+^ ions exhibited a slow decay time of 0.67 ms, suggesting limited alternative pathways for de-excitation besides radiative recombination. However, with an increasing Er^3+^ concentration, the Yb^3+^ decay became progressively more rapid, as evidenced by the shorter decay times. The increasing decay rate with higher Er^3+^ concentration highlighted the probability of energy transfer from Yb^3+^ to Er^3+^, which rises as more Er^3+^ ions are introduced into the lattice. Higher Er^3+^ concentrations increased the likelihood of an excited Yb^3+^ ion finding an Er^3+^ ion and facilitated a nonradiative energy transfer. This efficient energy transfer mechanism can enhance Er^3+^ luminescence and make Yb^3+^ a valuable sensitizer for Er^3+^ in applications where Er^3+^ emission at 1550 nm is desired. The energy transfer efficiency (orange data points, Figure 2g) increased as the Er^3+^ concentration rose and reached nearly 80% at the highest Er^3+^ concentrations. This efficient energy transfer mechanism made Yb^3+^ an effective sensitizer for Er^3+^ in such co-doped systems because it enabled energy absorbed by Yb^3+^ to be channeled into 1550 nm Er^3+^ emissions.

The nature of the energy transfer can be determined by analyzing the Yb^3+^ (donor) decay time profile using the Inokuti–Hirayama model (I–H model).^36,37^ The decay intensity *I*(*t*) of the donor in the I–H model can be expressed by eq 3,

3 $$I\left(t\right)=I_{0}\exp\left[-\frac{t}{\tau_{0}}\Gamma_{s}{\left(\frac{t}{\tau_{0}}\right)}^{3/s}\right]$$

where *t* is the time after excitation, *I*_0_ is the initial intensity at *t* = 0, τ_0_ is the intrinsic decay time of the donor emission in the absence of acceptors (Er^3+^ ions), Γ_s_ provides information on the probability of the energy transfer (proportional to the Er^3+^ concentration), and *S* is the multipolar interaction parameter based on the type of interaction: 6 for dipole–dipole interaction (Förster-type), 8 for dipole–quadrupole interaction, and 10 for quadrupole–quadrupole interaction. The temporal evolution of the ^2^F_5/2_ → ^2^F_7/2_ Yb^3+^ emission was fitted to eq 2 with τ_0_ = 0.665 ms (the decay time obtained for the Yb^3+^ solely doped sample Gd_2_O_3_:0.025Yb^3+^), considering values of *S* = 6, 8, 10. The best agreement between the Yb^3+^ emission decay time profiles and eq 2 was attained for *S* = 6 (red curves in Figure 2f). This result indicated that an electric dipole–dipole interaction was likely the dominant mechanism in the energy transfer between the Yb^3+^ and Er^3+^ ions. Figure S7a,b shows the decay of Er^3+^ luminescence as the concentration of Er^3+^ ions increased in Gd_2_O_3_:*y*Er^3+^, with a common shortening of the decay times as the concentration rises. The observed trend of decreasing decay times with higher Er^3+^ ion concentration was a typical effect in rare-earth-doped materials, where increased ion density facilitates energy transfer between Er^3+^ neighboring ions and reduces the emission lifetime. The temperature dependence of luminescence properties is shown in Figures S8a–d, S9a,b, and S10a–d. The electron paramagnetic resonance (EPR) study was conducted to analyze the local structure. The first derivative of the absorption spectrum was recorded as a function of applied magnetic induction. The following formula determines the position of the EPR line: *g*_eff_ = 7144773·*f*_rez_(GHz)/*B*_rez_(mT), where *g* is the Zeeman splitting factor, and the integrated EPR signal intensity was calculated as the area under the absorption curve. The EPR magnetic susceptibility was described by the Curie–Weiss eq 4.

4 $$\chi_{\mathrm{EPR}}=\frac{C}{T-T_{\mathrm{CW}}}$$

where *T*_CW_ is the Curie–Weiss temperatur, and *C* is a constant. The study material had three paramagnetic elements composed of Er^3+^ and Yb^3+^ ions doped Gd_2_O_3_: gadolinium, erbium, and ytterbium. They all provided an EPR signal; however, Er^3+^ and Yb^3+^ were observed only in the helium temperature range due to the electron configuration. Gd^3+^ ions with a quenched magnetic orbital moment (*L*= O) provided an EPR signal over the entire temperature range. The change in intensity in the 1536 nm emission was caused by the transition from the ^4^I_13/2_ level to the ^4^I_15/2_ level due to a change in the excitation source. Figure 2h,i presents the spectra for the Gd_2_O_3_ compound doped with different Er^3+^ and Yb^3+^ ion concentrations. A broad (in the full range of magnetic induction of ∼1.2 T) intense signal at *g*_eff_ = 2.177 originated from Gd^3+^ ions. This typical EPR signal was from strongly interacting Gd^3+^ ions. The dopants (Er^3+^ and Yb^3+^) provided a signal in the induction range up to *B* < 0.6 T and, therefore, occurred in superposition; the dominant signal came from Gd^3+^ ions and was not visible in the EPR spectrum. However, their effect on the integrated magnetic properties of the material was noticeable. Minor changes in the integrated intensity of the EPR signal were observed as the concentrations of Er^3+^ and Yb^3+^ ions increased. This outcome indicated that the Gd^3+^ sites were occupied by Er^3+^ and Yb^3+^ ions. The integrated EPR signal intensity and Curie–Weiss equation parameters fit for selected concentrations of Er^3+^ and Yb^3+^ ions are presented in Figure S11. The dynamic light scattering analysis showed the average particle size and zeta potential for the GOYE and GOYE@APTES nanoparticles, as presented in Figure S12a,b. Table S1 summarizes the absorbance/emission wavelength, IQE (%)-NIR-IIb region, and application of the different NIR-II probes. We also measured the IQE (%)-NIR-IIb region for Y_2_O_3_:Yb^3+^,Er^3+^ (YOYE), and NaYF_4_:Yb^3+^/Er^3+^ (NaYFYE) nanophosphors and compared them with our GOYE system, as shown in Figure S13. Our GOYE system’s IQE(%) was way higher than the oxide- and fluoride-based nanophosphors, whose IQE(%) was only around 2% and 5% respectively. It can be clearly observed that our nanoprobe Gd_2_O_3_:0.025Yb^3+^,0.01Er^3+^ (GOYE) shows the highest IQE at 22.8% in the NIR-IIb region after excitation with a 980 nm laser. The relationship of the 976 nm laser power intensity with the NIR-II signal intensity of the GOYE lanthanide system is shown in Figure S14. The graphs in Figure S14a,b confirm that the NIR-II signal intensity also increased with the increase in laser power intensity, and the NIR-II signal intensity showed a linear relationship with the increase in laser power intensity.

### NIR-II Imaging and Penetration Depth Analysis of GOYE and GOYE@APTES

The NIR-II imaging setup is shown in Figure 3a, where an 808 and 980 nm laser was used to excite the NIR-II probes (GOYE and GOYE@APTES). An InGaAs camera was utilized to capture the NIR-II images of the samples with a 1100 nm long-pass (LP) filter. Figure 3b presents the image of a stacked chicken breast tissue set up on top of the Eppendorf tubes containing the GOYE and GOYE@APTES samples. The penetration depth of our NIR-II probes was around 1 cm within breast tissue. The NIR-II images of the lanthanide powders were obtained using an NIR-II IVIS Ninox 640II instrument with a laser of 808 nm, a digital gain of 2 dB, and an exposure time of 1 ms, as shown in Figure S15a–d. Five different concentrations of APTES to coat the GOYE sample were tried: 40 μL of APTES/30 mg of GOYE powder, 150 μL of APTES/30 mg of GOYE powder, 200 μL of APTES/30 mg of GOYE powder, 250 μL of APTES/30 mg of GOYE powder, and 300 μL of APTES/30 mg of GOYE powder. Figure S16a indicates the NIR-II image for the different concentrations of the APTES-synthesized GOYE samples at 1 mg. Although GOYE had the highest NIR-II signal intensity, it agglomerated at the bottom of the Eppendorf tube after dispersion with deionized (DI) water. The different APTES coatings displayed satisfactory dispersibility in water, with 250 μL of APTES coating around GOYE powder. The quantitative assessment (normalized NIR-II intensity plot) also showed the same trend for the NIR-II signal intensity (Figure S16b). The NIR-II signal intensity after stacking chicken breast tissues on the Eppendorf tubes containing our samples is shown in Figure S17a, where the NIR-II probes showed a penetration depth of up to 9 mm after λ_ex_ = 808 nm. Moreover, the coating sample (GOYE@APTES) demonstrated better penetrability than the uncoated sample (GOYE). Figure S17b shows the NIR-II signal intensity after stacking chicken breast tissues on the Eppendorf tubes containing our samples, where our samples showed a penetration depth of up to 5 mm after λ_ex_ = 980 nm. At 980 nm laser excitation, the penetration depth is not that profound for both GOYE@APTES and the GOYE powder samples. Figure 3c represents the quantitative assessment of the relation between the NIR-II signal intensity and penetration depth and is in accordance with Figure S17a, where the NIR-II signal intensity decreases with an increase in the thickness of chicken breast tissues and shows maximum intensity until 9 mm tissue thickness. Figure 3d also shows the same trend as that in Figure S17b, and the NIR-II signal intensity remains bright up to 5 mm tissue thickness. GOYE@APTES shows NIR-II signal with a small difference in intensity compared to GOYE due to the effect of APTES coating, but it is not that prominent. The comparison between the relation between the NIR-II signal intensity and penetration depth of GOYE@APTES sample at λ_ex_ = 808 nm and λ_ex_ = 980 nm excitation shows that with λ_ex_ = 980 nm, the decrease in NIR-II signal intensity is abrupt due to the absorption of water at this wavelength. However, for λ_ex_ = 808 nm, the decrease is more gradual. So it confirms that λ_ex_ = 808 nm provides better penetration depth than λ_ex_ = 980 nm (Figure 3e). The penetration depth study setup is shown in Figure S18a,b, along with the setup to measure the thickness of chicken breast tissues. The NIR-II signal decreased gradually from 0 to 11 mm. The images demonstrated that the NIR-II signal could be observed at almost 9 mm for λ_ex_ = 808 nm and 5 mm for λ_ex_ = 980 nm tissue thickness, which confirmed the high penetration depth of the GOYE@APTES at λ_ex_ = 808 nm.

**Figure 3:**
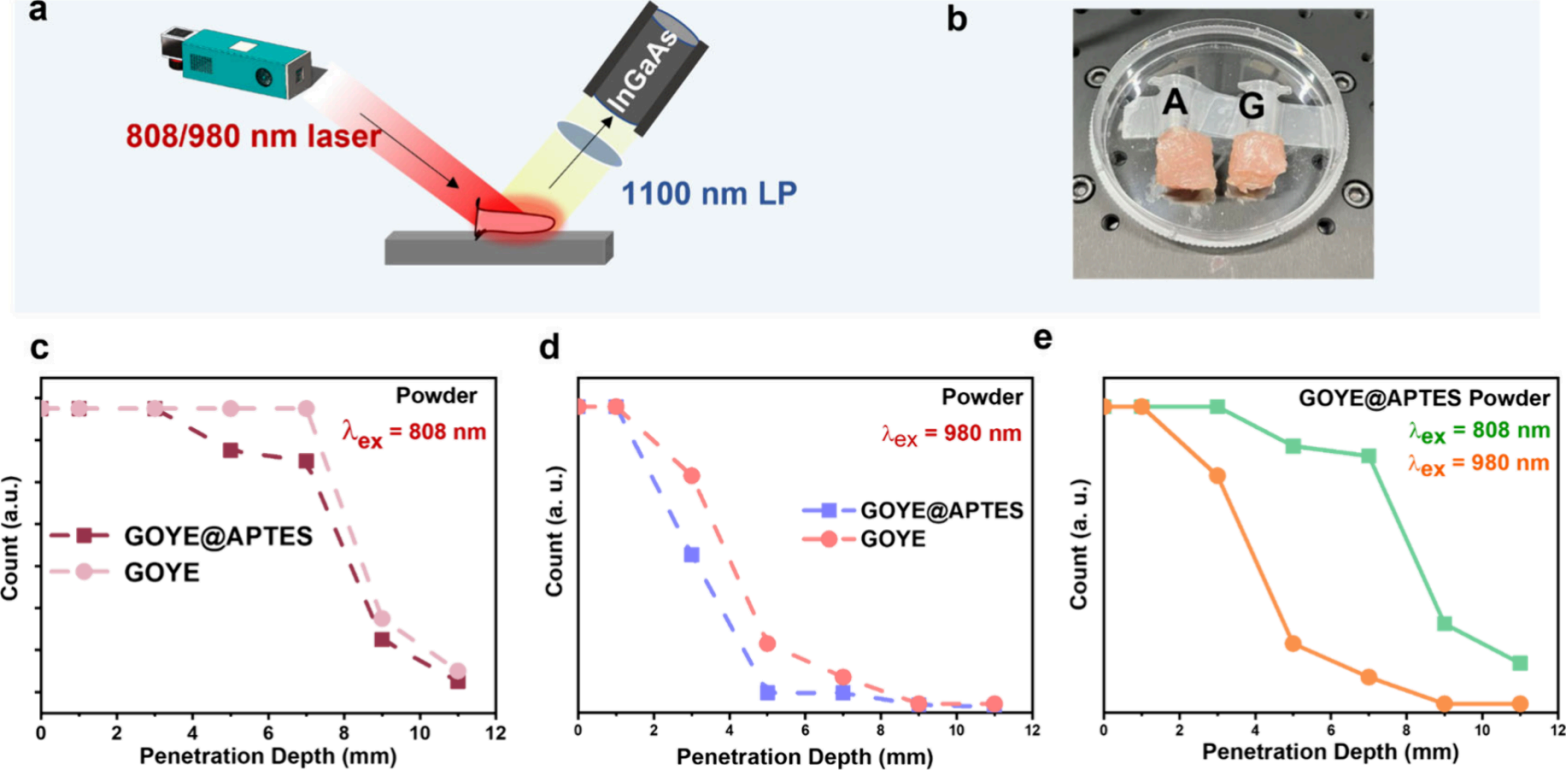
(a) Experimental setup for NIR-II imaging with a 1100 nm LP filter. (b) Stacked chicken breast tissue set up on top of the Eppendorf tubes containing the GOYE (G) and GOYE@APTES (A) samples. Quantitative assessment of fluorescence penetration depth in the NIR-II region under simulated tissue (digital gain = 3 dB and exposure time = 30 ms) with chicken breast tissue of different thicknesses (0, 1, 3, 5, 7, 9, and 11 mm), for (c) GOYE and GOYE@APTES powder samples at λ_ex_ = 808 nm and (d) GOYE and GOYE@APTES powder samples at λ_ex_ = 980 nm and (e) comparative analysis of fluorescence penetration depth for GOYE@APTES powder samples for λ_ex_ = 808 and 980 nm.

Figure S19 illustrates the NIR-II signal detected after placing chicken breast tissues of varying thicknesses on Eppendorf tubes containing the GOYE and GOYE@APTES samples dispersed in phosphate-buffered saline (PBS) solution. GOYE and GOYE@APTES demonstrated satisfactory NIR-II signal intensity even when dispersed in PBS. However, the GOYE@APTES sample had less aggregation and showed uniform dispersion in PBS. The NIR-II signal gradually decreased as the tissue thickness increased from 1 mm to 5 mm. Notably, the NIR-II signal remained detectable even at a thickness of 5 mm, which highlighted the remarkable penetration capability of the GOYE@APTES sample. In comparison, the uncoated GOYE sample exhibited significant aggregation at the bottom of the Eppendorf tubes, negatively affecting the uniformity of its NIR-II signal intensity. Conversely, the GOYE@APTES sample maintained a more consistent and uniform NIR-II signal, which suggested that the APTES coating enhanced its optical properties and mitigated the aggregation issue. These findings emphasized the superior performance of the GOYE@APTES sample in terms of penetration depth and signal stability, which makes it a promising candidate for biomedical imaging applications where deep tissue penetration is essential. The observed differences between the two samples underscored the importance of surface functionalization in improving the optical characteristics of nanomaterials for advanced imaging technologies.

### Biocompatibility Analysis of GOYE and GOYE@APTES

Figure 4a,b represents the cell viability plot for the NeHepLxHT (normal liver cells) and Mahlavu (cancer cells) treated with GOYE and GOYE@APTES. The liver cells were chosen for *in vitro* studies, as nanoparticles tend to accumulate in the liver after iv injection.^38^ Five different concentrations were made using the serial dilution technique (0.25, 0.125, 0.0625, 0.03125, and 0.015625 mg/mL) to treat the cells for 72 h. GOYE and GOYE@APTES, both samples, showed a similar trend of cell viability for NeHepLxHT cells, where increasing concentration of the lanthanide samples reduced the cell viability to 73% and 64%, respectively. The highest cell viability obtained for both samples was greater than 80% for the NeHepLxHT and Mahlavu cells. Both lanthanide samples had minor cytotoxicity toward the NeHepLxHT normal liver cells and Mahlavu cancer cells. The *P value was calculated concerning the GOYE@APTES samples. Figure 4c presents the hemolysis test for the GOYE and GOYE@APTES samples after serial dilution using PBS with concentrations of 250, 125, 62.5, 31.3, and 15.65 μg/mL. To measure the hemolysis rate, eq 5 was utilized,

5 $$\%\mathrm{hemolysis}=\frac{A_{s}-A_{0}}{A_{\infty }-A_{0}}$$

where *A*_s_ is the absorbance of the samples at 540 nm, *A*_0_ is the absorbance of the negative control at 540 nm, and *A*_∞_ is the absorbance of the positive control at 540 nm. The GOYE sample showed its highest hemolysis rate, at 6.65 ± 0.1% for a 125 μg/mL blood concentration. By contrast, the maximum hemolysis rate for the GOYE@APTES system was notably lower, recorded at 0.66 ± 0.01% for the 15.65 μg/mL blood concentration. Additionally, the hemolysis rates for GOYE@APTES remained well below 4% at the lower end, which were significantly reduced compared with that of the uncoated GOYE system. The APTES coating played a crucial role in enhancing the biocompatibility of GOYE, limiting its hemolytic activity and reducing the potential for red blood cell damage. Figure 4d presents the hematoxylin and eosin (H & E) staining of the nude mice organs after iv injection of 10 mg/mL GOYE@APTES. The brain, spleen, liver, heart, lung, and kidney tissues showed no histological change after treatment with the GOYE@APTES sample.

**Figure 4:**
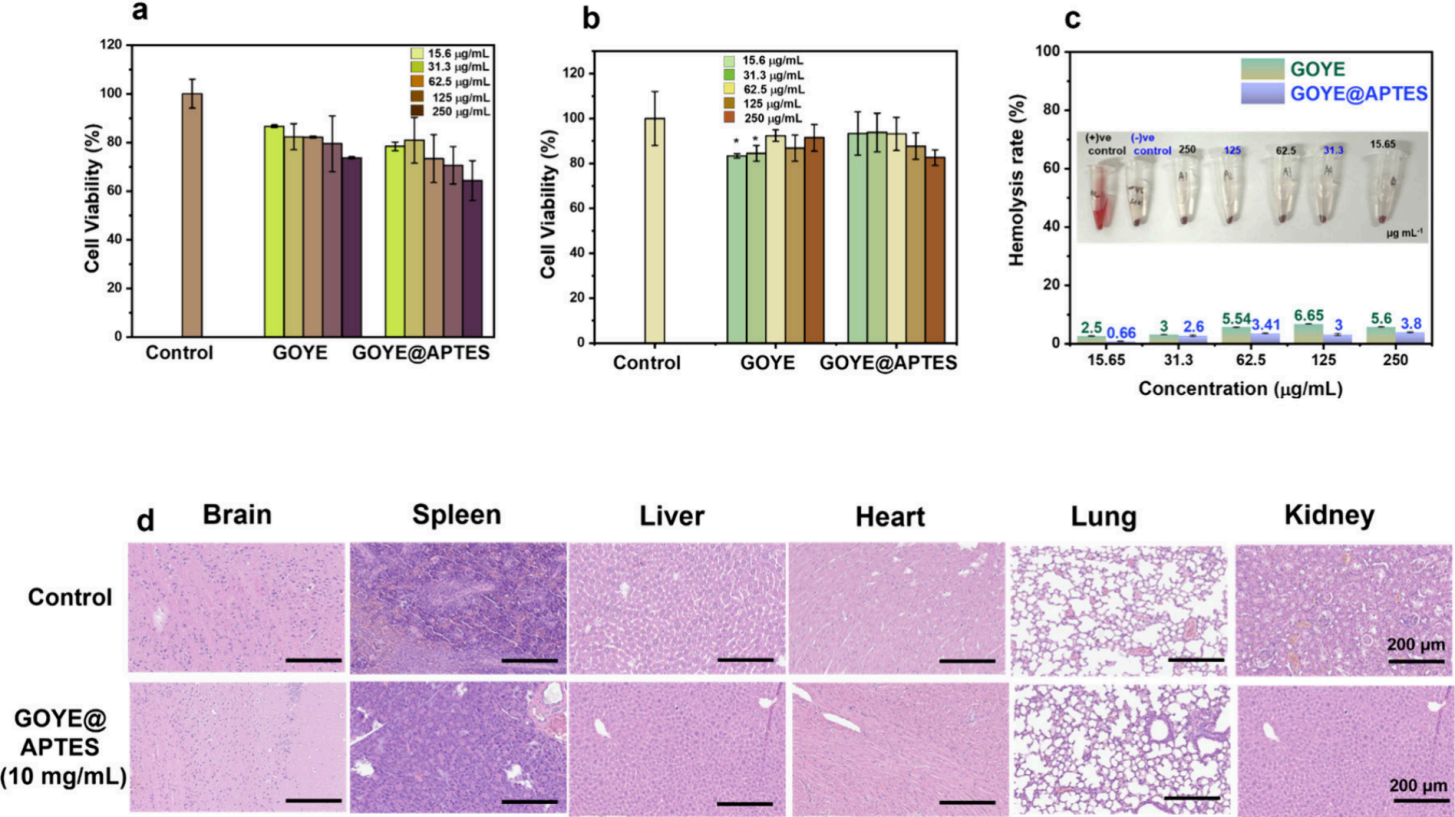
Cell viability plot of GOYE and GOYE@APTES samples for (a) NeHepLxHT (normal liver cells). (b) Mahlavu cells (cancer cells) with significance *P < 0.05. (c) Analysis of hemolysis rate for GOYE and GOYE@APTES systems (the inset figure shows the hemolysis test of the GOYE@APTES (250 μL) samples after serial dilution of both samples using PBS. (d) H & E staining of the organs of the nude mice treated with 10 mg/mL concentration of GOYE@APTES nanoparticle.

### MRI Analysis

Figure 5a shows the T1 and T2 mappings of the GOYE and GOYE@APTES images and confirms the presence of MRI signals in the samples. The concentrations of the GOYE and GOYE@APTES samples were 17.2, 8.6, 4.3, 2.15, and 1.075 mM. The slope of the fitted curve corresponded to the longitudinal (r1) and transverse (r2) relaxivity, as shown in Figure S20a. The T1 and T2 relaxivity of the GOYE was 0.0009 and 0.3262 mM^–1^ s^–1^, respectively. The low values of r1 could be due to the low concentration and the size of the GOYE nanoparticles.^17^ The higher value of r2 suggested that our GOYE system could be used as a T2 contrast agent instead of a well-recognized T1 contrast agent. The slope of the fitted curve corresponded to the T1 and T2 relaxivity, as shown in Figure S20b. The r1 and r2 of GOYE@APTES were 0.0048 and 0.8558 mM^–1^ s^–1^, respectively. The results were similar to those of the uncoated GOYE nanoparticle. The higher value of r2 could stem from the larger average size of the Gd_2_O_3_ nanoparticles. As the average size of the Gd_2_O_3_ nanoparticles increased, it led to a higher value of the r2/r1.^39,40^ The values of r2/r1 for both GOYE and GOYE@APTES exceed 150 in Figure 5b, which confirms that our nanoprobe could possibly be used as a T2 contrast agent, making organ images appear more darker,^41^ and this phenomenon is due to the larger size of our GOYE@APTES nanoprobe around 140 nm (Figure S12a) and also the shape of our nanoprobe.^42^ In this study, 200 μL of GOYE@APTES was administered to mice via an iv injection through the tail vein. The scanning region was centered on the liver, and imaging was performed 10 min postinjection. Figure 5c,d shows the T1- and T2-weighted MRI scans, respectively. Following the iv injection of GOYE@APTES and taken up by NOD-SCID mice, the T1-weighted images revealed increased brightness in the liver, along with visualization of the medulla and renal pelvis in the kidney. In the T2-weighted images, the contrast between the renal pelvis and the renal vertebral body was enhanced after injection.

**Figure 5:**
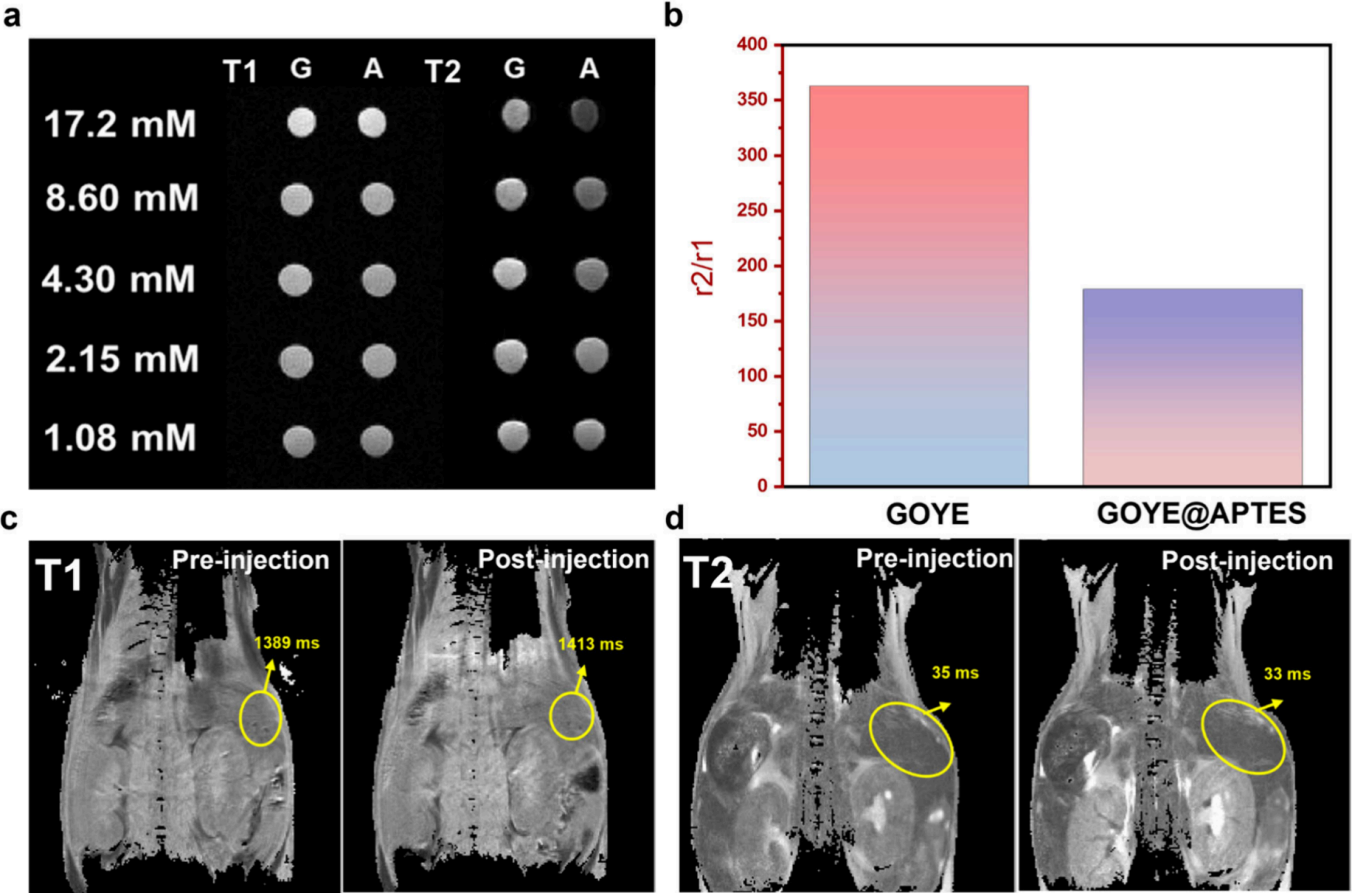
(a) T1- and T2-weighted MRI images of the GOYE and GOYE@APTES samples. (b) r2/r1 ratio comparison for GOYE and GOYE@APTES powder samples. (c) T1 mapping of the echo time (TE) intensity of the NOD-SCID mice after 200 μL of GOYE@APTES iv injection. (d) T2 mapping of the TE intensity of the NOD-SCID mice after 200 μL of GOYE@APTES iv injection.

### Comparison of NIR-II *in Vivo* Imaging with λ_ex_ = 808/980 nm Laser

The scheme for showing the setup for *in vivo* mice NIR-II imaging is shown in Figure 6a, where an InGaAs NIR-II camera captured the images using a 1300 nm LP filter. Figure 6b shows the NIR-IIb images *in vivo* in NUDE mice after the iv injection of GOYE@APTES (10 mg/mL). The control mice without GOYE@APTES showed no NIR-IIb intensity. Figure 6b shows the images under λ_ex_ = 808 nm and 980 nm are adjusted to a clearer exposure time, and the brightness of the two images is observed to reveal the superficial tissue development. The quantitative measurement of the NIR-II signal intensity is shown in Figure S21, where 980 nm laser excitation shows superior NIR-II signal intensity and brightness compared with 808 nm laser excitation. Figure 6c corroborates the organ imaging of the NUDE mice after the iv injection of GOYE@APTES (10 mg/mL PBS) with an exposure time of 4000 ms and λ_ex_ = 808 nm. The liver, large intestine (LI), and urinary bladder (UB) of the mice are easily visible. The signal-to-background ratio (SBR) for the region of interest (ROI) on the liver showed a value of 2.6 with a full width at half-maximum (fwhm) of 12 mm (Figure 6d). Figure 6e also shows the NIR-IIb signal intensity with the same concentration of iv injection and exposure time under 980 nm laser excitation in NUDE mice with only the liver of the mice visible. The SBR for the ROI on the liver showed a value of 3.3 with an fwhm of 21 mm (Figure 6f). Both 808 and 980 nm laser excitations have their own merits and demerits. Although the SBR is more for 980 nm laser excitation, the fwhm is broader compared to 808 nm laser excitation and NIR-IIb intensity. Moreover, the 808 nm laser excitation showed more deep-seated organs like the large intestine, with a narrow fwhm, validating the chicken penetration depth results in Figure 3. This proves the importance of 808 nm laser excitation for bioimaging purposes.

**Figure 6:**
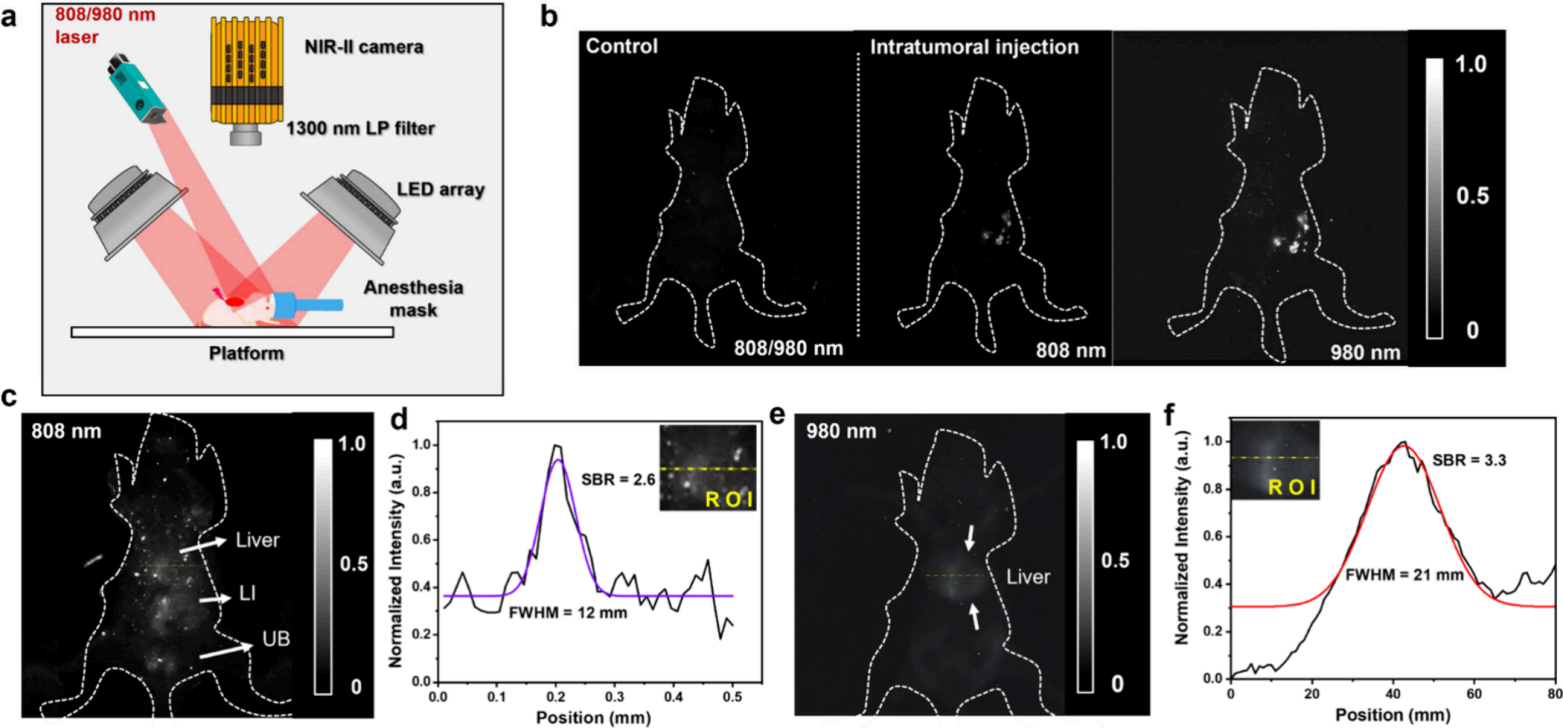
(a) Scheme showing the setup for *in vivo* mice NIR-II imaging using λ_ex_ = 980 nm and 1300 nm LP filter. (b) Subcutaneous NIR-II *in vivo* imaging (digital gain = 2 dB, exposure time = 3500 ms and 2500 ms, for λ_ex_ = 808 and 980 nm, respectively) of the NUDE mice model after the injection of GOYE@APTES (10 mg/mL PBS). (c) Subcutaneous NIR-II *in vivo* imaging (digital gain = 2 dB, exposure time = 4000 ms, and λ_ex_ = 808 nm), showing the different organs (liver, large intestine (LI), and urinary bladder (UB) of the NUDE mice model. (d) SBR and fwhm calculation for the region of interest (ROI) on the liver in (c). (e) Subcutaneous NIR-II *in vivo* imaging (digital gain = 2 dB, exposure time = 4000 ms, and λ_ex_ = 980 nm), showing only the liver of the NUDE mice model. (f) SBR and fwhm calculation for the region of interest (ROI) on the liver in (e).

## Conclusions

Gd_2_O_3_ nanoparticles co-doped with varying Yb^3+^ and Er^3+^ ion concentrations were successfully synthesized to improve the energy transfer efficiency and emission intensity. XRD and SAED analysis verified that the synthesized GOYE system maintained a pure phase with no detectable impurities, which indicated a high-quality crystal structure. XPS also revealed the formation of stable Gd–O bonds and verified the presence of Yb^3+^ and Er^3+^ ions within the nanoparticle structure. HRTEM was used to assess the morphology of the synthesized nanoparticles, which revealed a uniform shape and an average particle size of around 30 nm. This consistent particle size is advantageous for applications with critical particle homogeneity such as bioimaging. PL and QY measurements confirmed the optical properties of the GOYE nanoparticles, which displayed strong NIR-IIx and NIR-IIb emission and the highest intensity at 1530 nm and corresponded to Er^3+^ ions. The total quantum yield for the GOYE sample was 41.1% in the NIR region, and a significant quantum yield of 22.8% was explicitly observed in the NIR-IIx and NIR-IIb regions. These results underscored the nanoparticles’ potential as effective agents for bioimaging, where emission intensity in the NIR-II range is beneficial for deep-tissue imaging applications. A vital feature of the GOYE system is its efficient energy transfer, which increases as the concentration of Er^3+^ ions rises. At the highest Er^3+^ concentrations, the energy transfer efficiency reached nearly 80%, demonstrating that Yb^3+^ is an effective sensitizer. This efficiency was due to the capacity of Yb^3+^ to maximize the absorption of 980 nm energy and to transfer it effectively to Er^3+^ ions, which resulted in enhanced emissions from 1450 to 1580 nm. The APTES coating provided a uniform layer around the GOYE nanoparticles, which resulted in an average particle size of approximately 28 nm. Enhanced NIR-II signal intensity was detected in NIR-II imaging using an InGaAs camera, which underscored the imaging potential of the GOYE@APTES system. *In vitro* biocompatibility and *in vivo* bioimaging results further validated this system’s low toxicity and capabilities as a contrast agent; strong NIR-II signals were captured with IVIS imaging, which supported the potential of GOYE@APTES for applications in NIR-II and MRI imaging. These findings confirmed the downshifting energy transfer of the Yb^3+^-Er^3+^ system and the feasibility of GOYE@APTES as a promising NIR-II and MRI imaging platform and offered enhanced biocompatibility and signal quality for early disease detection. The multifunctional imaging capability makes GOYE@APTES a promising candidate for future biomedical applications.

## Materials and Methods

### Materials

Gadolinium­(III) nitrate hexahydrate (Gd­(NO_3_)_3_·6H_2_O, 99%) was provided by ACROS. Citric acid monohydrate (C_6_H_8_O_7_·H_2_O) was purchased from Riedel-de Haen. Polyethylene glycol (PEG)-20000 (H­(OCH_2_CH_2_)_*n*_OH) was purchased from Fluka. Erbium­(III) nitrate pentahydrate (Er­(NO_3_)_3_·5H_2_O, 99.8%) and ytterbium­(III) nitrate pentahydrate (Yb­(NO_3_)_3_·5H_2_O, 99.8%) were provided by Sigma-Aldrich. *N*,*N*-Dimethylformamide (DMF, 99.8%) and (3-aminopropyl)­triethoxysilane (APTES, 98%) were purchased from Sigma-Aldrich. Yttrium chloride hexahydrate (YbCl_3_.6H_2_O), Erbium chloride hexahydrate (ErCl_3_·6H_2_O), ytterbium chloride (YCl_3_.6H_2_O), yttrium acetate hydrate (Y­(CH_3_CO_2_)_3_·*x*H_2_O), ytterbium acetate hydrate Yb­(CH_3_CO_2_)_3_·*x*H_2_O, erbium acetate hydrate Er­(CH_3_CO_2_)_3_·*x*H_2_O Merck (Sigma-Aldrich), oleic acid, 1-octadecene, sodium hydroxide (NaOH), and ammonium fluoride (NH_4_F) were all purchased from Sigma-Aldrich.

### Sol–Gel Synthesis of Gd_2_O_3_:Yb^3+^,Er^3+^ Nanoparticles

The gel formation involved mixing Gd­(NO_3_)_3_, Yb­(NO_3_)_3_, and Er­(NO_3_)_3_ in 40 mL of DI water along with 2 g of citric acid. This mixture was stirred at 70 °C for 30 min. Subsequently, 2 g of PEG-20000 was added to the solution as a cross-linking agent, and vigorous mixing at 70 °C was continued until a wet gel formed. The wet gel was then dried in a vacuum oven at 80 °C for 24 h, and a white gel compound was created. In the next step, the gel compound underwent presintering at 250 °C for 5 h at a heating rate of 2 °C/min. This process yielded a precursor characterized by a brown, sticky consistency. The precursor was then ground for 1 min until it transformed into a brown powder. This brown powder was subsequently placed in a furnace for sintering at a higher temperature of 800 °C for 3 h at a heating rate of 2 °C/min. After cooling the powder to room temperature, the final Gd_2_O_3_:*x*Yb^3+^,*y*Er^3+^ white luminescent powder was obtained.

### Sol–Gel Synthesis of Y_2_O_3_:Yb^3+^,Er^3+^ Nanoparticles

The upconverting nanophosphors were synthesized by utilizing a Pechini sol–gel method. The process began with the preparation of an aqueous precursor solution by dissolving YCl_3_ (1 wt %), YbCl_3_ (5 wt %), and ErCl_3_ (0.2 wt %) in 20 mL of deionized water. This mixture was stirred continuously for 1 h to ensure complete dissolution and homogeneity. Subsequently, citric acid was introduced into the solution at a concentration of 10 wt %. Stirring was continued for an additional 1 h to facilitate the formation of a stable metal-citrate complex. The resulting homogeneous precursor solution was then transferred to a crucible and dried in an oven at 120 °C for 12 h. The dried material was collected as a solid mass and subjected to a final heat treatment, where it was annealed in a furnace at 800 °C for 3 h. This calcination step served to decompose the organic residues and crystallize the product, yielding the final white ceramic nanophosphors.^43^

### Co-Precipitation Synthesis of NaYF_4_:Yb^3+^/Er^3+^ Nanoparticles

The co-precipitation synthesis involved high-temperature dissolving the precursor solutions of Y­(CH_3_CO_2_)_3_ (3.2 mL, 0.2 M), Yb­(CH_3_CO_2_)_3_ (0.72 mL, 0.2 M), and Er­(CH_3_CO_2_)_3_ (0.8 mL, 0.02 M) using a two-neck flask and 80 °C heating for reaching crystallization. Subsequently, a mixture of oleic acid (6 mL) and 1-octadecene (14 mL) was introduced. This combination was heated to 120 °C with constant stirring in an oil bath until a clear solution was achieved through the complete dissolution of the crystalline material. The temperature was then elevated to 170 °C and maintained for 1 h to facilitate the formation of complexes capped with oleic acid. After this reaction, the solution was allowed to cool to 45 °C. In a separate preparation, methanolic stock solutions of NaOH (2 mL, 1 M) and NH_4_F (7.9 mL, 0.4 M) were combined and then added to the precursor mixture. To evaporate the methanol, the resulting mixture was heated incrementally to 70 °C, 90 °C, and finally 110 °C, holding at each temperature for 20 min. This was followed by a 10 min vacuum application, assisted by a liquid nitrogen cold trap, which prompted a color transition from a turbid yellow to a transparent orange. The reaction was then progressed under an inert argon or nitrogen atmosphere by heating the solution to 305 °C for 1.5 h. After this period, the mixture was cooled within the heating mantle to 45 °C, yielding a solution with an amber color. For purification, the nanoparticles were isolated by adding a nearly 1:1 mixture of cyclohexane and ethanol to the solution, followed by centrifugation at 7600 rpm for 6 min to pellet the product and discard unreacted supernatant residues. This washing process was repeated for a second time. The final purified nanoparticles were redispersed in cyclohexane (3 mL per tube) to create a transparent yellow colloidal dispersion.

### APTES Coating on GOYE Material

First, 30 mg of GOYE powder was placed in 10 mL of solution containing 1 mM NaOH and stirred for 6 h. The mixture was centrifuged at 7000 rpm for 10 min using ultrapure water and dried overnight. The GOYE powder, functionalized with the −OH group, was first dispersed in DMF for 15 min. Subsequently, 40 μL of APTES was added, and the mixture was stirred for 5 h. Afterward, the mixture was washed three times alternately with DMF and DI water using centrifugation and dried overnight to obtain the GOYE@APTES powder.

## Supplementary Material



## Data Availability

The data supporting this study’s findings are available from the corresponding authors upon reasonable request.

## Notes

Detailed experimental procedures of structural characterizations, fluorescence quantum yield, temperature-dependent PL decay curves, DLS and FTIR analysis, NIR-II imaging, and penetration depth (PDF)
