# Supplementary material for: Gd2O3 Doped with Yb3+/Er3+ for Boosted Downshifting Pathway in NIR-IIb Region and Exploring the Dynamics of MRI/NIR-II Imaging in the Nanophosphor
Source: ACS Appl Mater Interfaces. 2025 Oct 22;17(44):60789–801. doi: 10.1021/acsami.5c14860 (PMC12598694; doi:10.1021/acsami.5c14860)
Supplement: Supplementary file 1 [file am5c14860_si_001.pdf]

Supporting Information

Gd_2O_3 doped with Yb^{3+}/Er^{3+} for Boosted Downshifting Pathway in NIR-IIb Region and Exploring the Dynamics of MRI/NIR-II Imaging in the Nanophosphor

Aishwarya Satpathy[a†], Tzu-Hsuan Liu[a†], Ting-Yi Su[a], Shiqi Yu[b], Wei Zhang[b], Datao Tu[b], Agata Lazarowska[c], Natalia Majewska[d,c], Grzegorz Leniec[e,f], Ewa Mijowska[e,f], Xueyuan Chen[b], Sebastian Mahlik[c], Ming-Hsien Chan[g], and Ru-Shi Liu[a*]*

[a]Department of Chemistry, National Taiwan University, Taipei 106, Taiwan.

[b]State Key Laboratory of Structural Chemistry, and Fujian Key Laboratory of Nanomaterials, Fujian Institute of Research on the Structure of Matter, Chinese Academy of Sciences, Fuzhou, Fujian 350002, China.

[c]Institute of Experimental Physics, Faculty of Mathematics, Physics and Informatics, University of Gdansk, Wita Stwosza 57, 80-308 Gdansk, Poland.

[d]Faculty of Chemistry, Adam Mickiewicz University, Uniwersytetu Poznańskiego 8, 61-614 Poznań, Poland.

[e]Department of Nanomaterials Physicochemistry, Faculty of Chemical Technology and Engineering, West Pomeranian University of Technology, Piastow Ave. 45, 70-311 Szczecin, Poland.

[f]Center for Advanced Materials and Manufacturing Process Engineering, West Pomeranian University of Technology, 70-310, Szczecin, Poland.

[g]Department of Biomedical Imaging and Radiological Sciences, National Yang Ming Chiao Tung University, Taipei 112, Taiwan.

Corresponding Authors

*Ming-Hsien Chan (mhchan@nycu.edu.tw)

*Ru-Shi Liu (rsliu@ntu.edu.tw)

Contents

1. Characterizations..2
2. Experimental procedures. 3
   2.1 Near-infrared (NIR)-II imaging 3
   2.2 Cytotoxicity analysis...3
   2.3 Animal studies 4
   2.4 Hemolysis assay..4
   2.5 NIR-II *in vivo* imaging...4
   2.6 MRI imaging..5
3. Supplementary figures. 6

1. Characterizations: We investigated the crystallographic phases of Gd_2O_3:xYb^{3+},yEr^{3+} lanthanide nanoparticles with varying concentrations through XRD using a Bruker D2 Phaser instrument. The Cu filament was the X-ray source with a wavelength (λ) of 1.5405 Å. The average particle size was determined through HRTEM using a JEOL JEM-2100F microscope. EDX analysis was also performed to assess the elemental composition of the nanophosphors. To analyze the functional groups on the Gd_2O_3:xYb^{3+},yEr^{3+} nanoparticle. The optical properties were validated using an FLS1000 spectrometer and a Hamamatsu Quantaurus QY plus instrument. The QY plus measurement was done for powder samples with λ_{ex} = 808 nm. Excitation spectra were obtained using a custom-made setup comprising an EQ99X laser-driven

Xe light source (Energetiq) coupled with a self-made grating monochromator operating between 250–1000 nm as the excitation source and an Andor SR-500i-D1 spectrometer equipped with a CCD camera (DU490A-1.7) operating in the 600–1700 nm wavelength as the luminescence detector. Temperature-dependent emission spectra were obtained using an Andor SR-500i-D1 spectrometer equipped with a CCD camera (DU490A-1.7) operating within the 600–1700 nm wavelength range. A xenon lamp with a monochromator and a laser with 980 nm emission served as excitation sources for temperature-dependent measurements. Decay profiles were established using a custom-made setup comprising a function generator to produce the square-shaped light pulses of duration 100 ms and fall time ca. 100 ns, a National Instrument Data Acquisition module, and an Avalanche Photodetector APD110C/M operated in 900–1700 nm. The temperature was controlled using a THMS600 Linkam stage temperature controller and an LNP95 liquid-nitrogen cooling pump system to achieve a 100–600 K range. EPR spectra were recorded at room temperature by using a conventional Bruker X-band ELEXSYS E500 spectrometer operating at 9.46 GHz. The magnetic induction reached 1.4 T. The average particle size was determined through HRTEM using a JEOL JEM-2100F microscope. FTIR was conducted to analyze the different functional groups on the Gd_2O_3:xYb^{3+},yEr^{3+} nanoparticle by using a Perkin Elmer (5102-62) instrument. Zeta potential and average diameter were assessed using a Zetasizer Nano (Malvern Co., Ltd).

2. Experimental procedures

2.1 Near-infrared (NIR)-II imaging: An in-house NIR-II IVIS Ninox 640II instrument was utilized to test different concentrations of Gd_2O_3:xYb^{3+},yEr^{3+} lanthanide powders. The NIR-II instrument was coupled with an 808 nm laser for excitation, with a power supply of 12 V direct current (DC). The exposure time for low gain ranged from 10 μs to 26.8 s, whereas that for high gain ranged from 100 μs to 26.8 s. The image-capturing camera was an InGaAs PIN-photodiode, and the frame rate was up to 120 Hz.

2.2 Cytotoxicity analysis: Cytotoxicity analysis was conducted using the Alamar blue assay, which is suitable for detecting fluorescence and visible light. Reaction outcomes were recorded by measuring absorbance at 570 and 600 nm wavelengths. The NeHepLxHT cell line (immortalized human hepatic cell) was cultivated in RPMI/DMEM (1:1) medium, and the Mahlavu cell line (hepatocellular carcinoma cell) was cultivated in DMEM medium supplemented with 10% fetal bovine serum and 1% triple antibiotic reagent. The cells were then incubated in a 5% CO_2 environment at 37 °C. Subsequently, the cells were seeded onto 96-well plates at a density of 2000 cells per well. After seeding, the cells were treated with varying concentrations (0.0625, 0.125, 0.25, 0.5, and 1 mg/mL) of GOYE and GOYE@APTES in the culture medium. Following a 24 h incubation period, the Alamar blue assay was used to evaluate cytotoxic effects.

2.3 Animal studies: *In vivo* experiments were conducted with the Affidavit of Approval of Animal Use Protocol Institutional Animal Care and Use Committee (IACUC) of National Yang Ming Chiao Tung University (no. 1130502). We used 6-week-old female non-obese diabetic mice (JAXTM NOD.CB17-Prkdcscid/NcrCrl; NOD-SCID) for the hemolysis test and MRI region detection and five-week-old male nude mice (Balb/c) as the diagnostic model for NIR-II imaging. To obtain the NIR-II images, 100 μL of a subcutaneous and intravenous injection of GOYE@APTES lanthanide sample was added into the above two mice strains (n = 3 per group).

2.4 Hemolysis assay: First, we collected fresh blood from NOD-SCID mice and centrifuged it at 1000–1500g for 5–10 min at 4 °C to pellet RBCs. We used the positive and negative control with all the lysed blood cells and the one with only phosphate-buffered saline (PBS) and mouse blood, respectively, to measure the absorbance at 540 nm. Then, we measured the absorbance for all the samples of GOYE and GOYE@APTES to determine the amount of lysed cells in the supernatant.

2.5 NIR-II penetration depth study: The detector, Ninox 1280, is a VIS-SWIR dual-band camera with an InGaAs sensing chip and a resolution of 1280 x 1024, with a response range of 0.6μm to 1.7μm. When refrigerated to -15°C, the dark current reading reaches no more than 2,000e/p/s. SNR measurements rely on the assumption of Gaussian noise. A widely used criterion(SNR ≥ 3)for the limit of detection (LOD), which means the signal is three times larger than the noise standard deviation. High Gain (HG) mode was applied in all image collections with readout noise 28 e^- , full well capacity 10,000 e^- , and dynamic range 47 dB.[1]

2.6 NIR-II *in vivo* imaging: An NIR-II IVIS Ninox 640II instrument was used to test the GOYE lanthanide powders' coated samples *in vivo* in the 6-week-old female non-obese diabetic mice (JAXTM NOD.CB17-Prkdcscid/NcrCrl; NOD-SCID) and 5-week-old Balb/c, nude male mice (IACUC number: 1130502). The in-house NIR-II instrument was consolidated with a 980 nm laser for excitation, with a power supply of 12 V DC. The exposure time for low gain ranged from 10 μs to 26.8 s, whereas that for high gain ranged from 100 μs to 26.8 s. The image-capturing camera was an InGaAs PIN-photodiode, and the frame rate was up to 120 Hz. The Ninox 1280 camera exhibits a quantum efficiency greater than 90% in the wavelength range of 1100–1300 nm.[1]

2.7 MRI imaging: First, the 1 mL GOYE@APTES samples were diluted to 250 μg/mL (highest concentration) with PBS. Serial dilution was used to prepare five more concentrations: 125, 62.5, 31.25, 15.625, and 7.8125 μg/mL. Then, polymerase chain reaction (PCR) tubes were collected, and 125 μL of solution from the above stock solution was poured into each PCR tube, followed by 125 μL of 1% agarose solution (1:1 ratio of sample to agarose solution). A Bruker Pharmascan 70/16 US MRI instrument was used to test the GOYE and 250 μL of GOYE@APTES samples. Variable repetition time (VTR) was used to map T1-weighted images with repetition time (TR) = 6000-3000-1500-1000-600-300-200-100 ms, average = 2, echo time (TE) = 7 ms, 4 slices, 1.5 mm slice thickness, matrix size = 256 × 256, field of view (FOV) = 70 × 70 mm, and scan time = 55 min. A multi-slice multi-echo (MSME) setup was

used to map T2-weighted images with TR= 3000 ms, average = 2, TE1= 8 ms, spacing = 8 ms, 10 echoes, 4 slices, 1.5 mm slice thickness, matrix size = 256 × 256, FOV = 70 × 70 mm, and scan time = 26 min. In addition to *in vitro* testing, this study also conducted MRI imaging tracking of samples in mice. The parameters used for *in vivo* liver MRI imaging in NOD-SCID mice were TR= 3000 ms, average = 2, TE1= 8 ms, spacing = 8 ms, 6 echoes, 2 slices, 1.5 mm slice thickness, matrix size = 256×256, FOV = 70 × 70 mm, and scan time = 26 min for MSME T2-weighted images. TR = 6000-3000-1500-1000-600-300-200-100 ms, averages = 2, TE = 7 ms, 2 slices, 1.5 mm slice thickness, matrix size = 256 × 256, FOV = 70 × 70 mm, and scan time = 55 min.

3. Supplementary figures

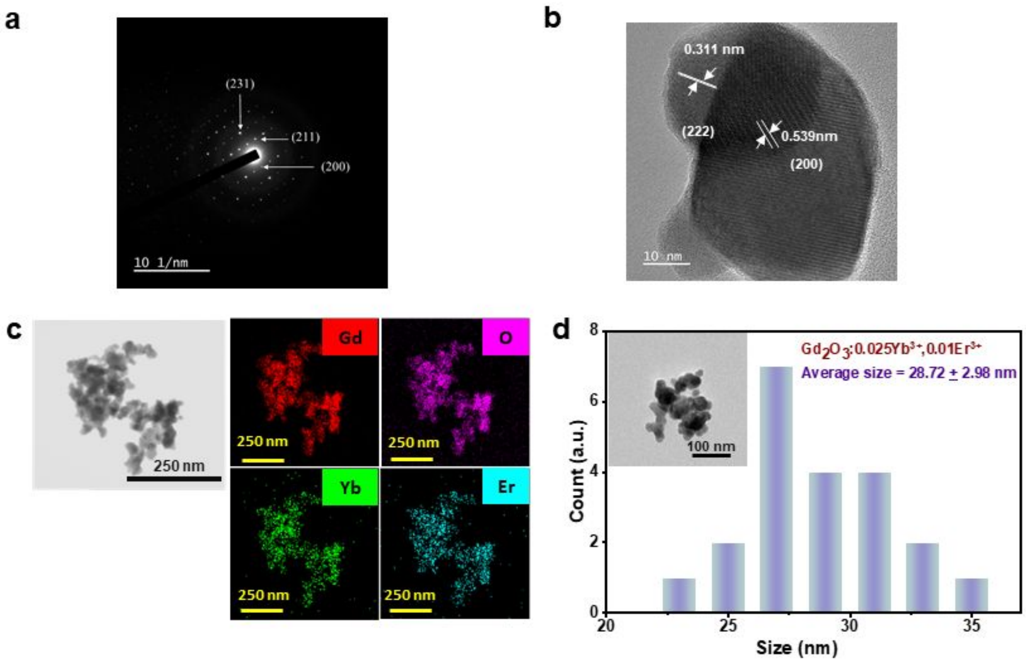

Figure S1. (a) SAED pattern of Gd_2O_3:0.025Yb^{3+},0.01Er^{3+} lanthanide system. (b, c) Dark-field lattice fringes for (222) and (200) planes, respectively, and the HRTEM image with EDS elemental mapping in the Gd_2O_3:0.025Yb^{3+},0.01Er^{3+} system. (d) Size distribution curve for Gd_2O_3:0.025Yb^{3+},0.01Er^{3+} nanoparticles.

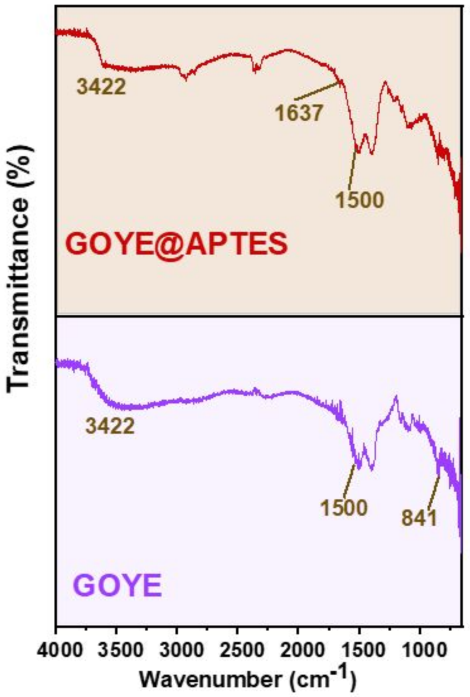

Figure S2. FTIR analysis of GOYE and GOYE@APTES lanthanide system.

The PLE spectra show the difference in excitation intensities for 808 nm and 980 nm. The 808 nm peak is more prominent in the case of GOYE, in comparison to YOYE and NaYFYE nanophosphors. The 980 nm peak is also higher for the GOYE sample in comparison to YOYE and NaYFYE nanophosphors. Although the 980 nm peak is of higher intensity than the 808 nm peak, 808 nm is more suitable for biological applications. Moreover, other materials do not show a distinct excitation peak at 808 nm, as can be observed in **Figure S3**.

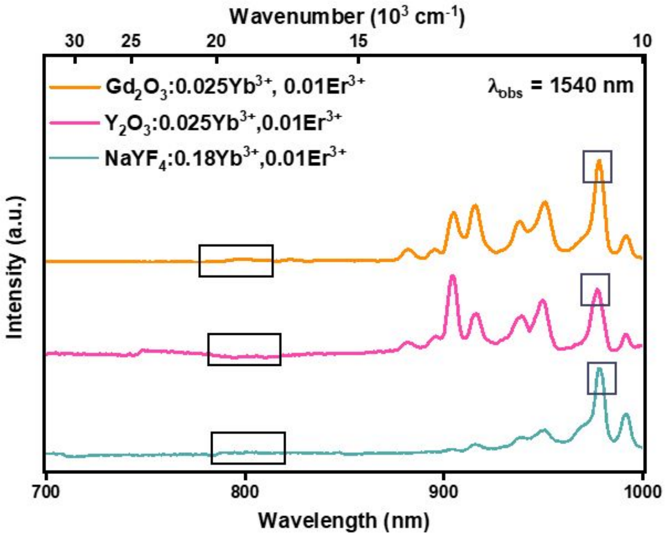

Figure S3. PLE spectra comparison for 808 nm and 980 nm excitation after λ_{em} = 1540 nm for GOYE, Y_2O_3:0.025Yb^{3+},0.01Er^{3+} (YOYE), and $NaYF_4$:0.18Yb^{3+},0.01Er^{3+} (NaYFYE) nanophosphors.

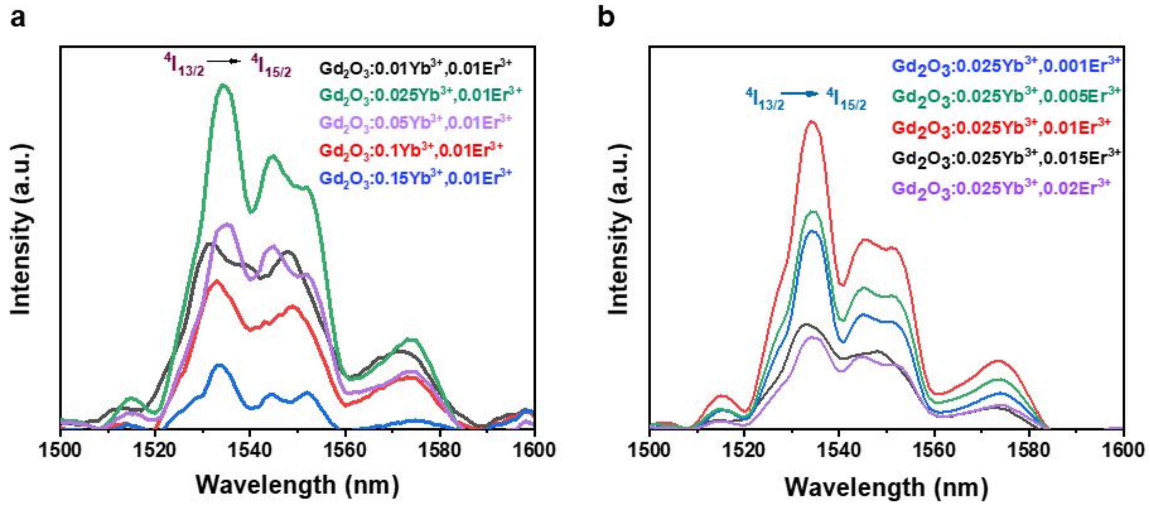

Figure S4. (a) PL spectra of Gd_2O_3:xYb^{3+},0.01Er^{3+} with x = 1%, 2.5%, 5%, 10%, and 15% after λ_{ex} = 980 nm. (b) PL spectra of Gd_2O_3:0.025Yb^{3+},yEr^{3+} with y = 0.1%, 0.5%, 1%, 1.5%, and 2% after λ_{ex} = 980 nm.

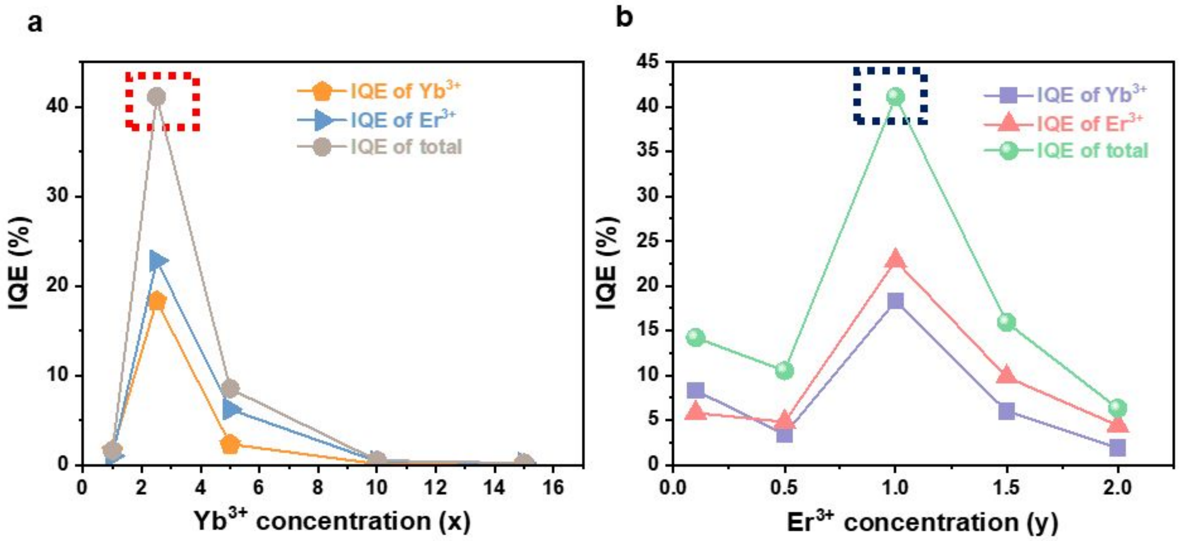

Figure S5. Internal quantum efficiency (IQE) analysis of powder samples with λ_{ex} = 980 nm for (a) Gd_2O_3:xYb^{3+},$0.01Er^{3+}$ with x = 1%, 2.5%, 5%, 10%, and 15%. (b) Gd_2O_3:$0.025Yb^{3+}$,yEr^{3+} with y = 0.1%, 0.5%, 1%, 1.5%, and 2%.

We wanted to check the highest-intensity emission of the $Gd_{2-x}O_3$:xYb^{3+} samples to determine whether 2.5% Yb^{3+} showed a good emission intensity after excitation with 915 nm because we chose this concentration to obtain the best emission intensity with Er^{3+} and Yb^{3+} doped samples. As observed in **Figure S6**, the excitation spectrum was measured at 915 nm to avoid overlapping with the 980 nm excitation. Additionally, the emission at about 1020 nm was inferred to be a slightly lower energy emission generated by doping, resulting in structural distortion. The intensity of the three samples x = 1, 2.5, 5% at about 976 and 1020 nm was not the same, which was also due to the different degrees of structural distortion.

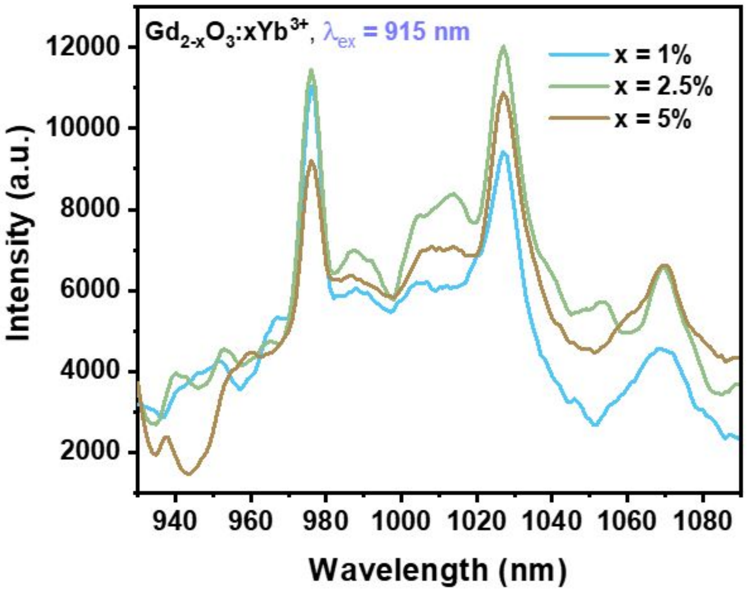

Figure S6. PL spectra of $Gd_{2-x}O_3$:xYb^{3+} samples after excitation with λ_{ex} = 915 nm.

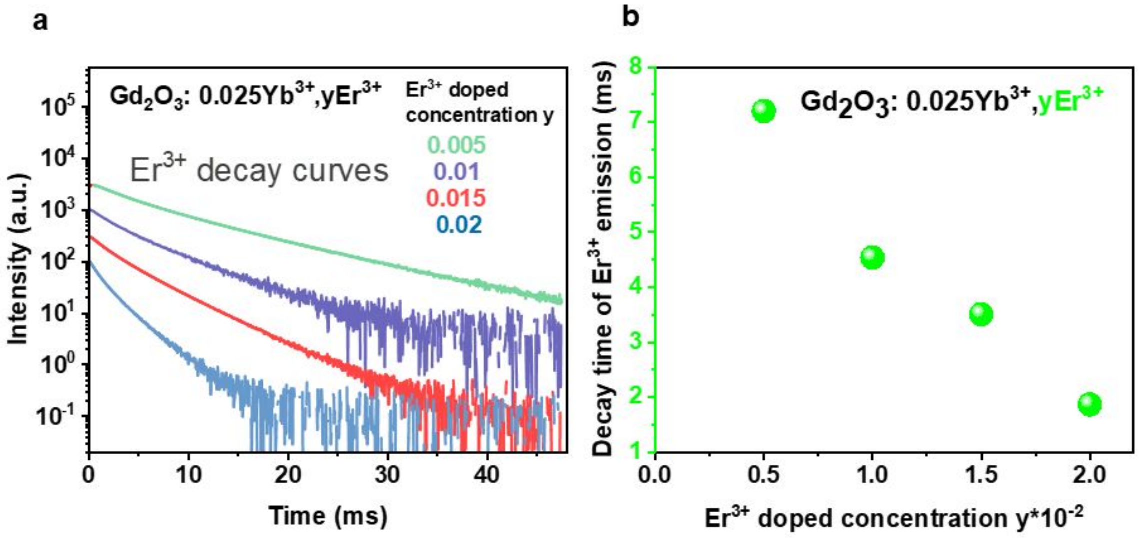

Figure S7. (a) Concentration-dependent decay profiles of Er^{3+} emission. (b) Average decay times of Gd_2O_3:0.025Yb^{3+},yEr^{3+} calculated using Equation 2.

Subsequently, we illustrated the temperature-dependent normalized PL spectra for various transitions in Gd_2O_3-doped samples under NIR excitation in **Figure S8**. **Figure S8**a shows the PL spectrum of the $^2F_{5/2}\rightarrow{}^2F_{7/2}$ transition in Gd_2O_3:0.025Yb^{3+} at 100–550 K with 915 nm

excitation. As the temperature rose, the emission lines broadened, and anti-Stokes phonon sidebands appeared on the high-energy side of the emission spectrum at 973 nm. **Figure S8**b displays the PL spectrum of the $^4I_{11/2}$→$^4I_{15/2}$ and $^4I_{13/2}$→$^4I_{15/2}$ transitions in Gd_2O_3:0.01Er^{3+} under 980 nm excitation within the same temperature range. **Figure S8**c shows the PL spectrum for the Yb^{3+} and Er^{3+} co-doped Gd_2O_3:0.025Yb^{3+},0.01Er^{3+} sample, where energy transfer occurred under 980 nm excitation. As the temperature increased, the emission lines broadened slightly, and anti-Stokes phonon sidebands related to Er^{3+} emerged on the high-energy side of the Er^{3+} emission spectrum, similar to those observed in Gd_2O_3:0.01Er^{3+}. **Figure S8**d presents the temperature-dependent integrated PL intensity for Gd_2O_3:0.025Yb^{3+} (orange points), Gd_2O_3:0.01Er^{3+} (green points), and Gd_2O_3:0.025Yb^{3+},0.01Er^{3+} (purple points) samples across the 100–550 K range. The observed temperature-dependent behavior of each sample provided insights into the energy-transfer dynamics and thermal stability of the PL emissions. The Yb^{3+}-doped sample (Gd_2O_3:0.025Yb^{3+}) showed an increase in the intensity of the $^2F_{5/}$→$^2F_{7/2}$ transition (950–1100 nm) with temperature up to around 300 K, followed by a gradual decline. Notably, the Gd_2O_3:0.025Yb^{3+} sample was also tested with 980 nm excitation. A similar temperature dependence of PL intensity was obtained; however, in such a measurement, we can examine only the luminescence spectrum above 1000 nm, which emitted the dominant emission peak at 980 nm. The emission intensity of Er^{3+} (1400–1650 nm) in Gd_2O_3:0.01Er^{3+} was relatively stable within the studied temperature range, and only a slight decrease in emission intensity (around 20 %) was observed with rising temperature. The temperature-dependent emission intensity of Er^{3+} in Gd_2O_3:0.025Yb^{3+},0.01Er^{3+} differed from that obtained for the solely doped Er^{3+} sample, Gd_2O_3:0.01Er^{3+}. In particular, anti-thermal quenching was observed as the emission intensity increased with rising temperatures up to 300 K and then started to decrease for higher temperatures. This finding was similar to that for the Yb^{3+}-doped sample, Gd_2O_3:0.025Yb^{3+}. Moreover, another important phenomenon is that the anti-thermal quenching is often governed by multiple temperature-dependent mechanisms rather than only phonon-

assisted excitation. One important contribution comes from the redistribution of population within the Stark-split sublevels of Er^{3+} ions. With rising temperature, higher-lying sublevels become increasingly occupied, and these can exhibit stronger radiative transition probabilities or more favorable involvement in upconversion processes, resulting in an apparent increase in emission or spectral modifications. A second contribution arises from changes in nonradiative pathways, such as Er→Yb back-transfer or Er–Er cross-relaxation. The rates of these processes are sensitive to temperature, and under certain conditions, elevated temperatures suppress them. This reduction in nonradiative losses maintains a higher population in the emitting state, thereby enhancing luminescence output. Overall, the enhancement of emission under heating can be more reasonably attributed to the combined effects of Stark-level redistribution and temperature-dependent relaxation dynamics, rather than to a model that assumes phonon involvement with constant energy-transfer efficiency.[2–4]

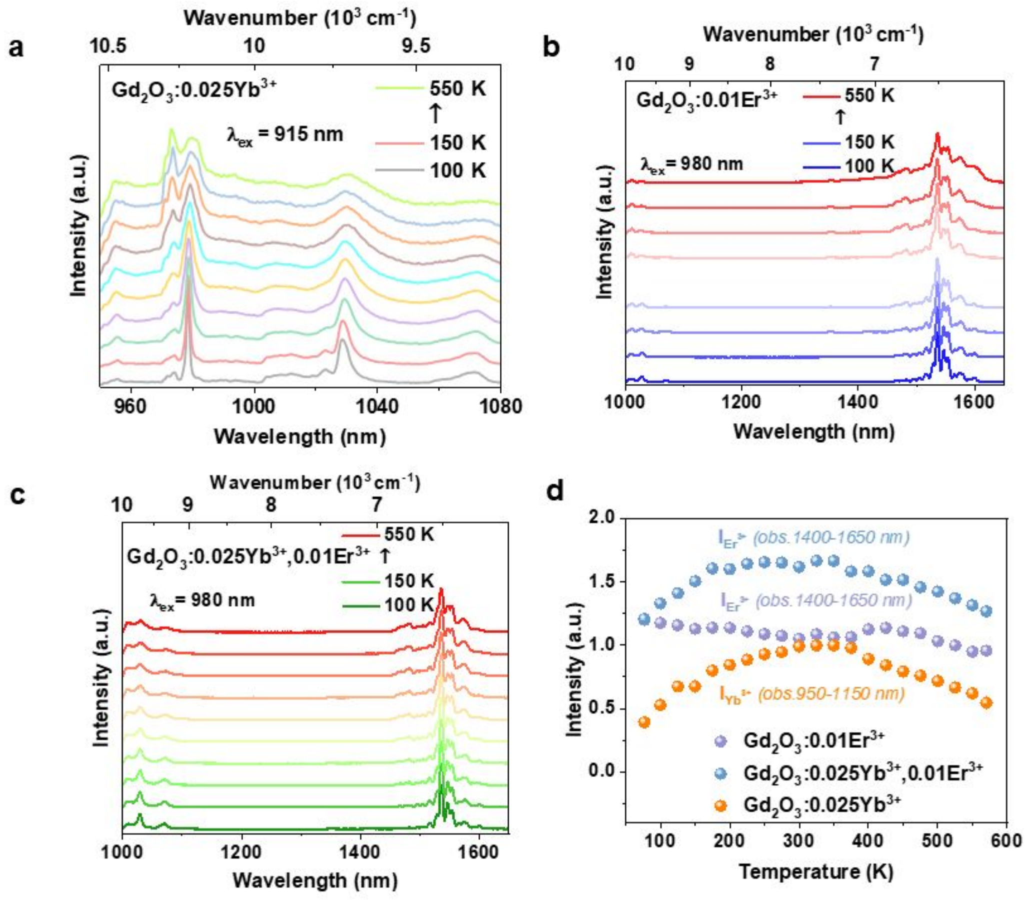

Figure S8. Temperature-dependent analysis: normalized PL spectra of (a) Gd_2O_3:0.025Yb^{3+}, (b) Gd_2O_3:0.01Er^{3+} , (c) Gd_2O_3:0.025Yb^{3+},0.01Er^{3+}; and (d) integrated PL intensity for all samples.

Figure S9a presents the temperature-dependent decay times of Yb^{3+} luminescence in Gd_2O_3 matrices, comparing samples co-doped with Er^{3+} (Gd_2O_3:0.025Yb^{3+},0.01Er^{3+}) and doped only with Yb^{3+} (Gd_2O_3:0.025Yb^{3+}). **Figures S10**a and **S10**b show the luminescence-decay profiles as a function of temperature, from which the presented decay times were obtained (by fitting a single-exponential function). As temperature increased from 100 K to 600 K, the trend of the changes in luminescence intensity in both samples was consistent. The temperature dependence of decay times differed from that of the emission intensity, increasing within 100–350 K from 0.38 ms to 0.69 ms and then saturating. Notably, the Yb^{3+}-only doped sample showed consistently longer decay times than the Er^{3+}, Yb^{3+} co-doped sample across the whole temperature range. This finding suggested that Er^{3+} co-doping enhanced nonradiative relaxation, most likely due to energy-transfer processes. The unusual behavior observed when the intensity and decay time of Yb^{3+} luminescence increased with increased temperature can be explained by several factors related to energy-transfer dynamics and the interaction between Yb^{3+} ions and their surrounding environment. This phenomenon may be attributed to a combination of factors, such as the thermal activation of radiative states and a reduction in nonradiative processes.

Moreover, **Figure S9**b displays the temperature-dependent decay times of Er^{3+} luminescence (by fitting a single-exponential function) in Gd_2O_3 matrices, comparing samples co-doped with Yb^{3+} (Gd_2O_3:0.025Yb^{3+},0.01Er^{3+}) and those doped solely with Er^{3+} (Gd_2O_3:0.01Er^{3+}). The luminescence-decay profiles used to obtain the decay times are shown in **Figures S10**c and **S10**d. The temperature dependence of decay times increased similarly for both samples. A slight difference was observed at high temperatures, where in the case of the sample co-doped

with Er^{3+} and Er^{3+} ions, the increase in the Er^{3+} luminescence decay time was slower than in the case of the sample doped only with Er^{3+}. When the decay time of luminescence increased and the intensity decreased with increased temperature, it implied a decrease in the radiative transition probability related to phonon interactions, symmetry-based selection rules, and balance between radiative and nonradiative decay pathways.

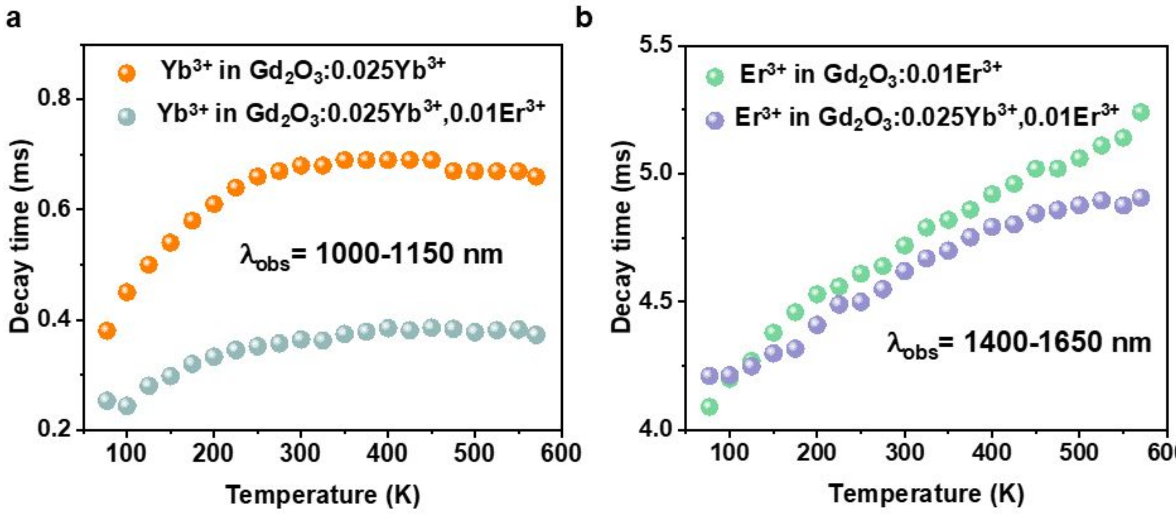

Figure S9. PL decay times for (a) Yb^{3+} luminescence observed at 1000–1150 nm and (b) Er^{3+} luminescence observed at 1400–1650 nm.

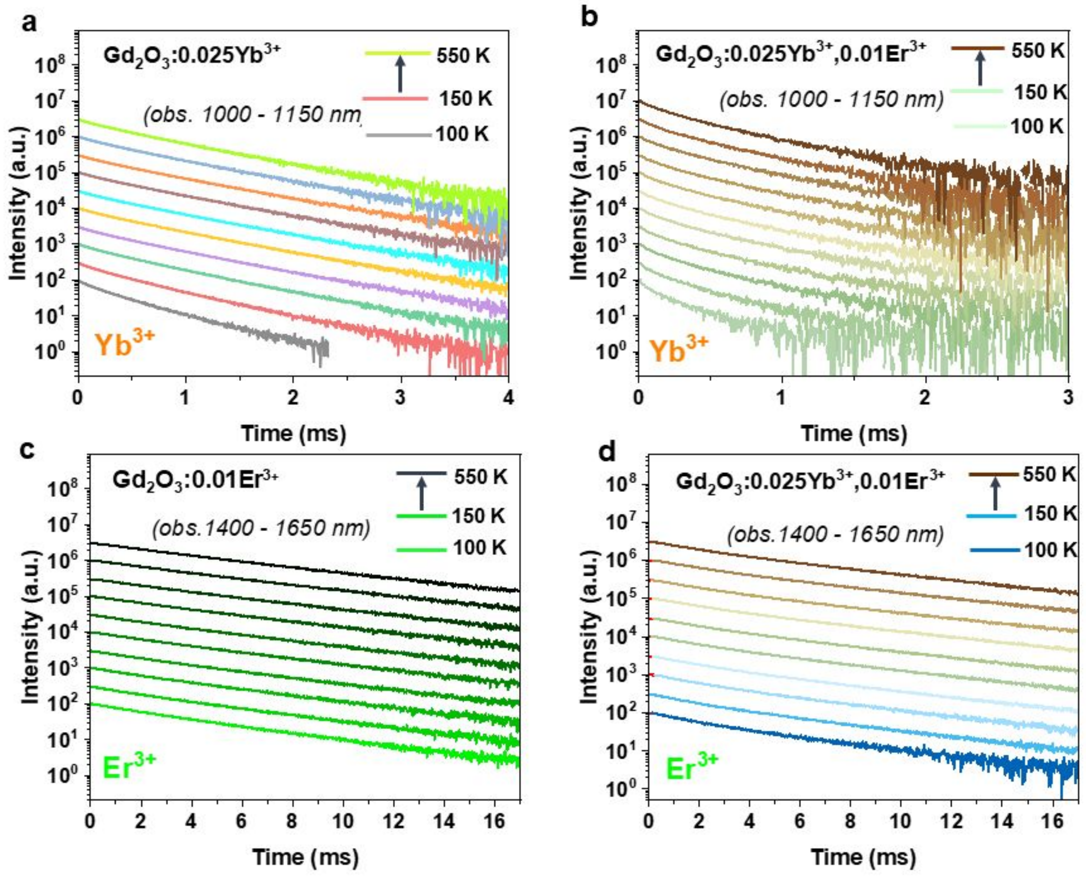

Figure S10. Temperature-dependent decay profiles of Yb^{3+} emission in (a) Gd_2O_3:0.025Yb^{3+} and (b) Gd_2O_3:0.025Yb^{3+},0.01Er^{3+}. Decay profiles of Er^{3+} emission in (c) Gd_2O_3:0.01Er^{3+} and (d) Gd_2O_3:0.025Yb^{3+},0.01Er^{3+}.

Small changes in the integrated intensity of the EPR signal were observed as the concentration of Er^{3+} and Yb^{3+} ions increased. This finding indicated that the Gd^{3+} sites were occupied by Er^{3+} and Yb^{3+} ions. Fitting the parameters of the Curie–Weiss equation indicated that strong antiferromagnetic interactions between Gd^{3+} ions dominate. The integrated EPR signal intensity and Curie–Weiss equation parameters fitted for the selected concentrations of Er^{3+} and Yb^{3+} ions are presented in **Figure S11**.

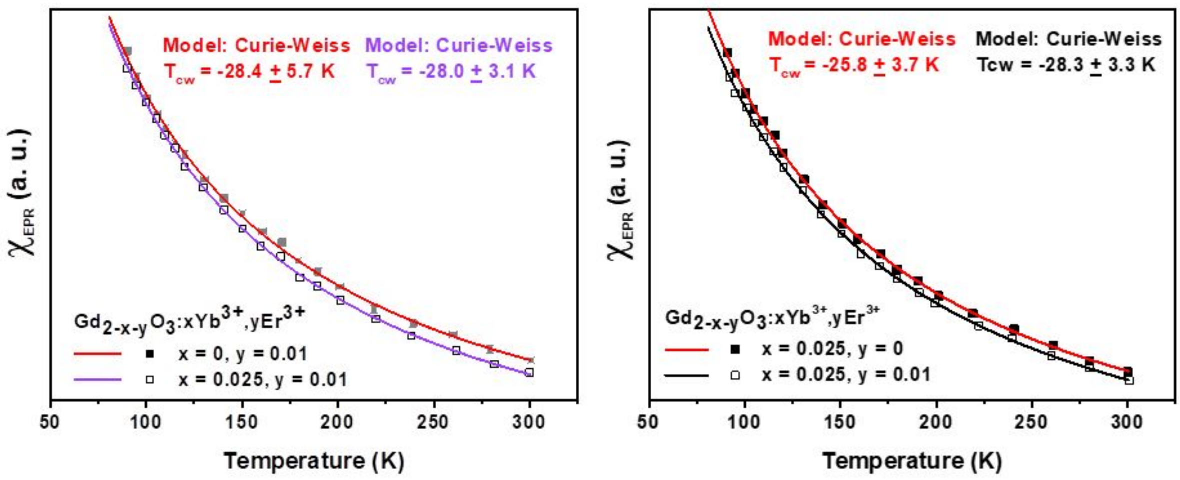

Figure S11. Temperature dependence of the EPR magnetic susceptibility within the nitrogen temperature range for Gd_2O_3 with selected Er^{3+} and Yb^{3+} ion concentrations.

GOYE had an average size of 105 ± 10 nm with an almost negative ζ potential of approximately 1.2 ± 0.8 mV (**Figures S12**a and **S12**b). Additionally, GOYE and APTES@GOYE had monodispersed peaks, indicating the prevention of aggregation. The average size of APTES@GOYE was around 132 ± 15 nm (**Figures S12**a); notably, the size of the nanoparticles increased owing to the high concentration of APTES and water surrounding

the nanoparticles. The positive ζ potential of 6.5 ± 2.4 mV (**Figures S12**b) was attributed to the amino group (-NH_2) of APTES and aligned with the FTIR results.

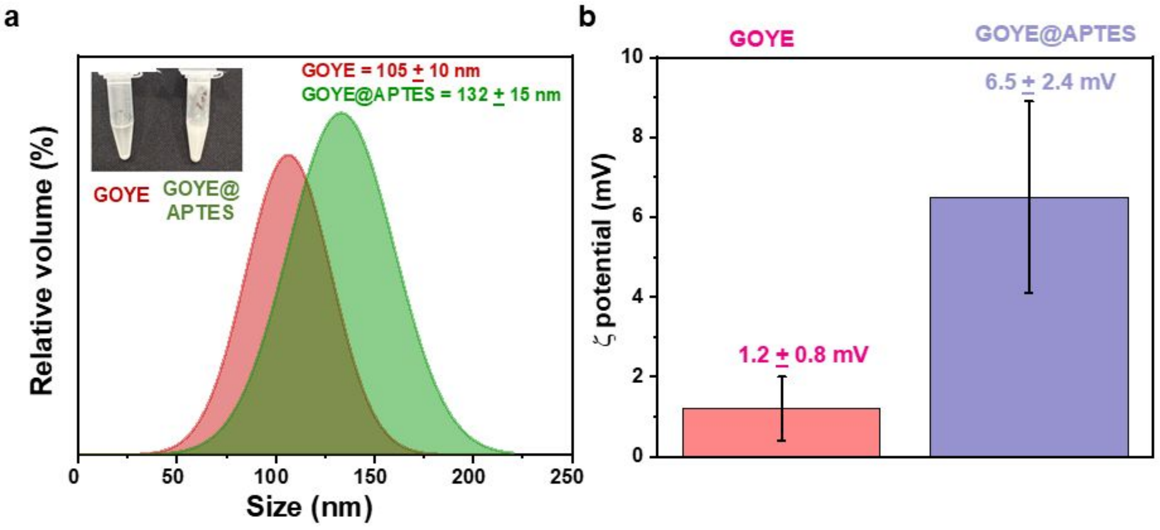

Figure S12. DLS analysis shows the (a) average size distribution and (b) zeta potential of the GOYE and GOYE@APTES lanthanide systems.

Table S1. Table summarizing various nanoprobes in comparison to our GOYE@APTES system.

**Nanoprobes**	**Absorbance/Emission (nm)**	**IQE (%)-NIR-IIb**	**Application**
**$Gd_2O_3:0.025Yb^{3+},0.01Er^{3+}$ (GOYE)**	**808/1530**	**22.8%**	**Organ imaging**
PbS/CdS quantum dots[5]	808/1500–1700	2–20%	Imaging of Vascular Regeneration
2TT-oC26B[6]	808/1500–1600	11.5%	Whole body imaging
FD-1080 J-Aggregates[7]	1360–1370/1500	0.0545%	Vascular imaging and hypertension monitoring
HL3[8]	750–1050/1550	0.005%	Cerebrovascular, lymph node, and whole-body imaging
HQL2[9]	710–1050/1500	0.002%	Tumor and vessels imaging

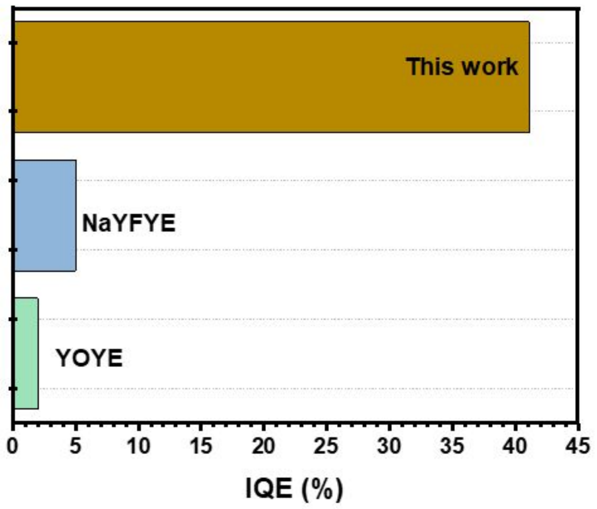

Figure S13. Comparison of IQE(%) for GOYE, NaYFYE, and YOYE nanophosphors.

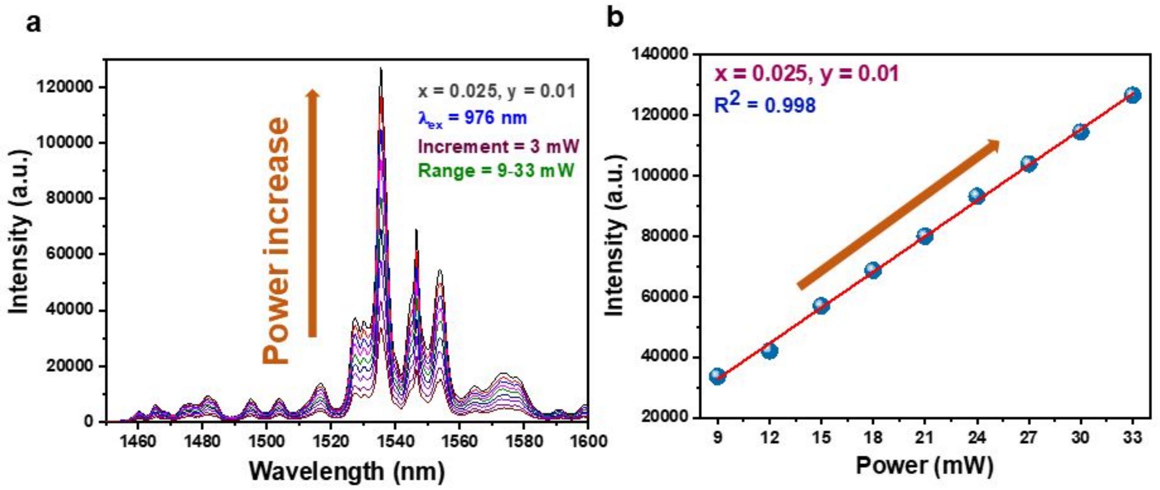

Figure S14. (a) Relationship of 976 nm laser power with NIR-II signal intensity for the GOYE sample (range = 9–33 mW, increment = 3 mW). (b) Plot of the linear relationship between 976 nm laser power (range = 9–33 mW, increment = 3 mW) with NIR-II signal intensity.

The NIR-II images of the lanthanide powders were obtained using the NIR-II IVIS Ninox 640II instrument with a laser of 808 nm, a digital gain of 2 dB, and an exposure time of 1 ms. **Figures S15**a **and S15**b confirm that the lanthanide powders had an excellent emission intensity with the 808 nm laser. The highest signal was obtained for the GOYE powder. These data

demonstrated that GOYE powder was a promising candidate for bioimaging and disease-diagnosis applications. **Figures S15**c and **S15**d provide a quantitative assessment of the NIR-II signal intensity, which aligned closely with the results obtained from NIR-II IVIS imaging. This consistency highlighted the reliability of the material's optical performance, emphasizing its capability as an effective NIR-II bioimaging agent. Thus, our study confirmed the material's suitability for applications requiring precise and high-quality imaging in biological contexts by validating the strong correlation between quantitative measurements and imaging outputs.

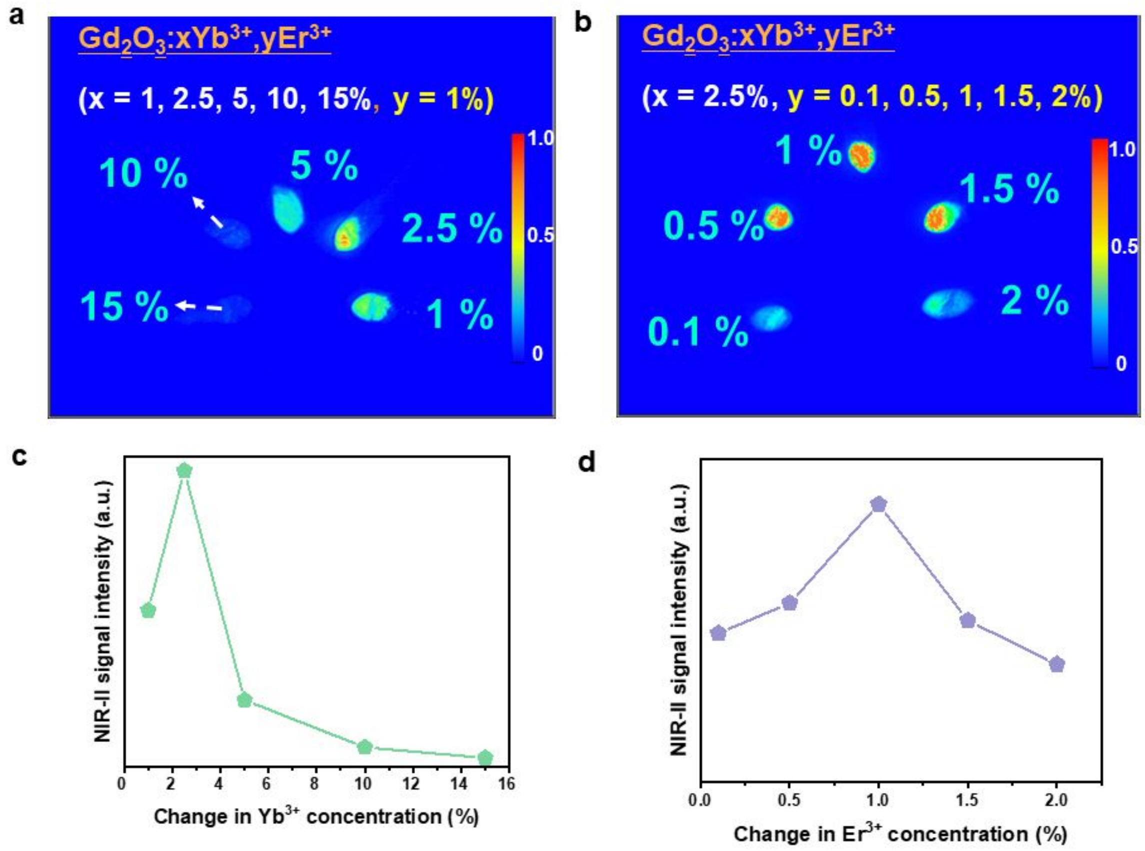

Figure S15. NIR-II imaging of (a) Gd_2O_3:xYb^{3+},yEr^{3+} with x = 1%, 2.5%, 5%, 10%, and 15%, y = 1% excited with an 808 nm laser, and (b) Gd_2O_3:xYb^{3+},yEr^{3+} with x = 2.5%, y = 0.1%, 0.5%, 1%, 1.5%, and 2% excited with an 808 nm laser. Quantitative assessment of the NIR-II signal intensity for (c) Gd_2O_3:xYb^{3+},yEr^{3+} with x = 1%, 2.5%, 5%, 10%, and 15%, y = 1%

excited with an 808 nm laser, and (d) Gd_2O_3:xYb^{3+},yEr^{3+} with x = 2.5%, y = 0.1%, 0.5%, 1%, 1.5%, and 2% excited with an 808 nm laser.

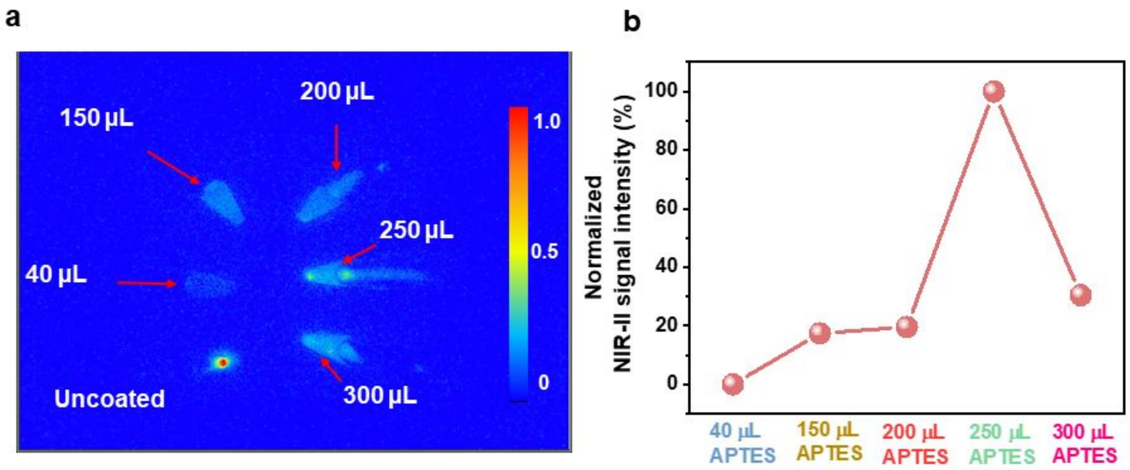

Figure S16. (a) NIR-II image (digital gain = 10 dB and exposure time = 50 ms) captured with an InGaAs camera after excitation with an 808 nm laser for APTES-coated GOYE samples in DI water. (b) Corresponding quantitative assessment of NIR-II signal intensity for different concentrations of APTES-synthesized coated GOYE sample in DI water.

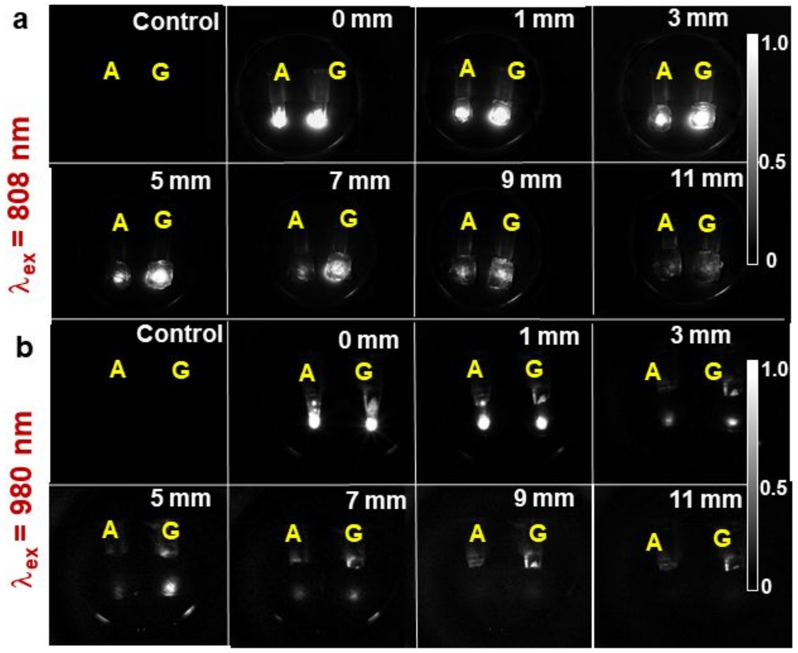

Figure S17. Fluorescence penetration depth in the NIR-II region under simulated tissue (digital

gain = 3 dB and exposure time = 30 ms) with chicken breast tissue of different thicknesses (0, 1, 3, 5, 7, 9, and 11 mm), for GOYE and GOYE@APTES powder samples, (where A denotes the GOYE@APTES sample, and G denotes the GOYE sample), under laser excitation of (a) 808 nm and (b) 980 nm.

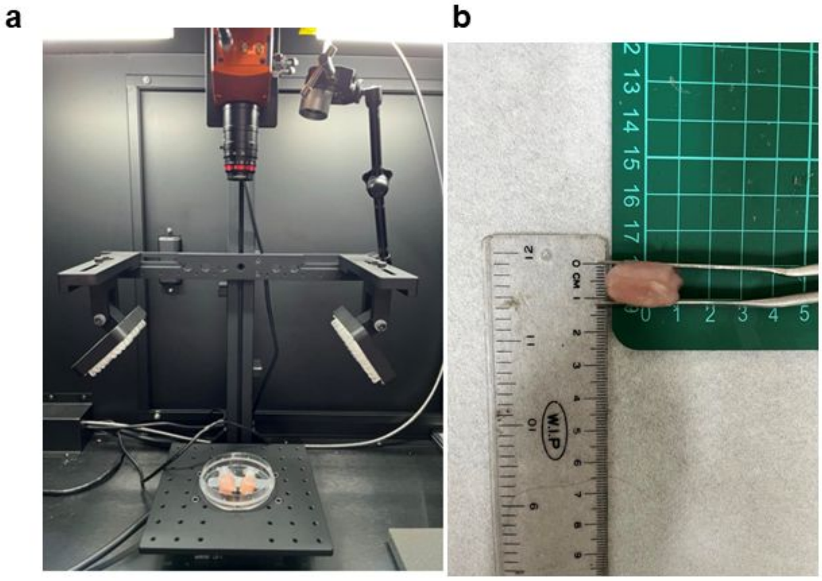

Figure S18. (a) A penetration depth study setup shows the chicken breast tissue stacking on the Eppendorf with the sample. (b) The thickness of the stacked chicken breast tissues was measured using a ruler on a measuring board.

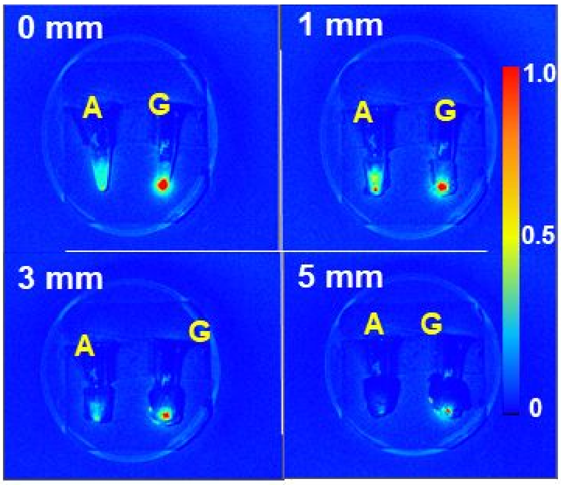

Figure S19. Fluorescence penetration depth in the NIR-II region under simulated tissue (digital gain = 5 dB and exposure time = 50 ms) with chicken breast tissue of different thicknesses (0, 1, 3, and 5 mm), for GOYE and GOYE@APTES samples (where A denotes the GOYE@APTES sample, and G denotes the GOYE sample) in PBS solution at λ_{ex} = 808 nm.

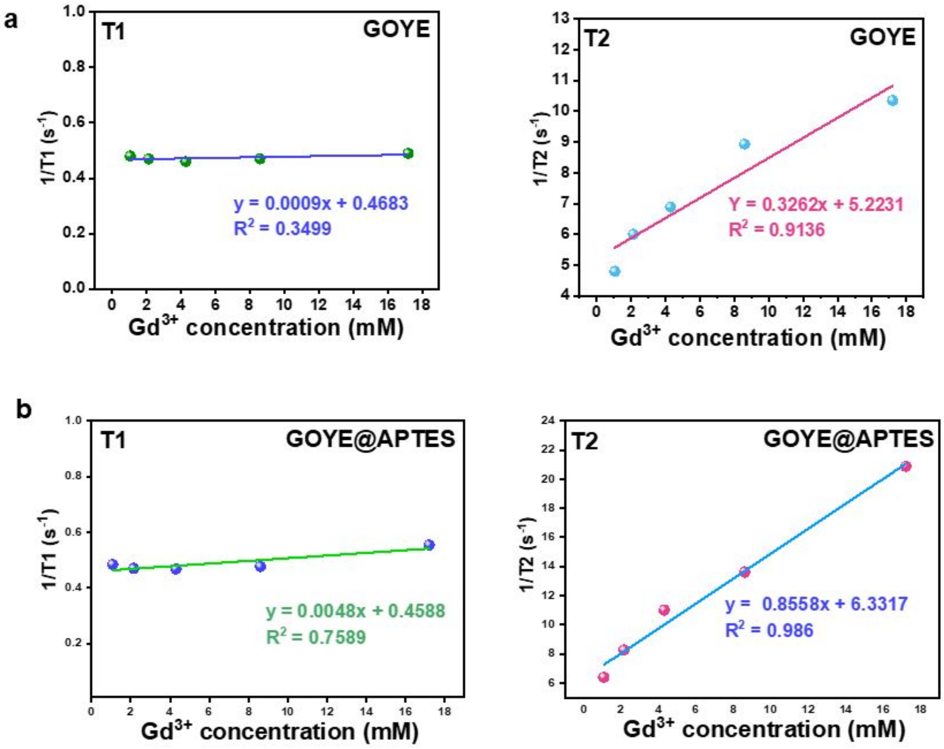

Figure S20. The graphs depict the linear correlation between (a) r1 and Gd^{3+} concentration, where r1 relaxivity is the slope of the curve of GOYE and r2 and Gd^{3+} concentration, and r2 relaxivity is the slope of the curve of GOYE; and (b) r1 and Gd^{3+} concentration, where r1 relaxivity is the slope of the curve of GOYE@APTES, and r2 and Gd^{3+} concentration, and r2 relaxivity is the slope of the curve of GOYE@APTES.

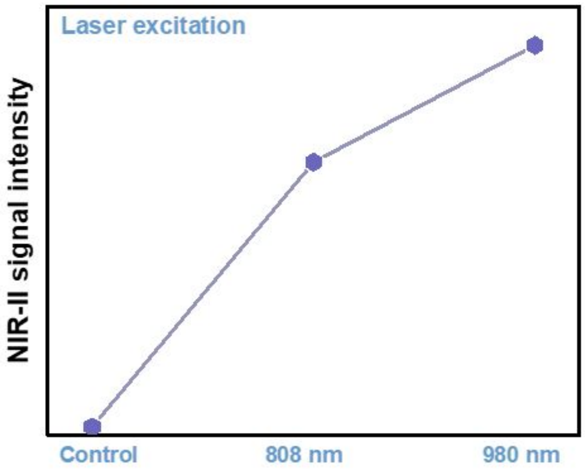

Figure S21. Comparative plot showing the change in NIR-II signal intensity after excitation with λ_{ex} = 808 nm and 980 nm.

References

(1) Photonics, R. "Ninox 1280. "https://www.raptorphotonics.com/products/ninox-1280/(accessed 2025-09-12).

(2) Huang, F.; Yang, T.; Wang, S. X.; Lin, L.; Hu, T.; Chen, D. Q. Temperature Sensitive Cross Relaxation Between Er Ions in Laminated Hosts: A Novel Mechanism for Thermochromic Upconversion and High Performance Thermometry. *J. Mater. Chem. C* **2018**, *6* (45), 12364–12370.

(3) Sukul, P. P.; Singh, Y.; Swart, H. Ultra-Wide Band Near-Infrared (NIR) Optical

Thermometry (12–673 K) Performance Enhanced by Stark Sublevel Splitting in Er^{3+} Ions near the First Biological Window in the $PbZr_{0.53}Ti_{0.47}O_3$:Er^{3+}/Yb^{3+} Phosphor. *Phys. Chem. Chem. Phys.* **2024**, *27* (1), 270–282.
(4) Wang, M.; Zhang, M.; Ding, S.; Hu, H.; Zhang, C.; Zou, Y. Effect of Energy Back Transfer from Er^{3+} to Yb^{3+} Ions on the Upconversion Luminescence of Er:NaYb$(MoO_4)_2$ and Yb,Er:NaBi$(MoO_4)_2$. *Front. Optoelectron* **2025**, *18* (1), 12.
(5) Ma, Z.; Zhang, M.; Yue, J.; Alcazar, C.; Zhong, Y.; Doyle, T. C.; Dai, H.; Huang, N. F. Near-Infrared IIb Fluorescence Imaging of Vascular Regeneration with Dynamic Tissue Perfusion Measurement and High Spatial Resolution. *Adv. Funct. Mater.* **2018**, *28* (36), 1803417.
(6) Li, Y.; Cai, Z.; Liu, S.; Zhang, H.; Wong, S. T. H.; Lam, J. W. Y.; Kwok, R. T. K.; Qian, J.; Tang, B. Z. Design of AIEgens for Near-Infrared IIb Imaging through Structural Modulation at Molecular and Morphological Levels. *Nat. Commun.* **2020**, *11* (1), 1255.
(7) Sun, C.; Li, B.; Zhao, M.; Wang, S.; Lei, Z.; Lu, L.; Zhang, H.; Feng, L.; Dou, C.; Yin, D.; Xu, H.; Cheng, Y.; Zhang, F. J-Aggregates of Cyanine Dye for NIR-II *In Vivo* Dynamic Vascular Imaging beyond 1500 nm. *J. Am. Chem. Soc.* **2019**, *141* (49), 19221–19225.
(8) Li, Y.; Liu, Y.; Li, Q.; Zeng, X.; Tian, T.; Zhou, W.; Cui, Y.; Wang, X.; Cheng, X.; Ding, Q.; Wang, X.; Wu, J.; Deng, H.; Li, Y.; Meng, X.; Deng, Z.; Hong, X.; Xiao, Y. Novel NIR-II Organic Fluorophores for Bioimaging beyond 1550 nm. *Chem. Sci.* **2020**, *11* (10), 2621–2626.
(9) Li, Q.; Ding, Q.; Li, Y.; Zeng, X.; Liu, Y.; Lu, S.; Zhou, H.; Wang, X.; Wu, J.; Meng, X.; Deng, Z.; Xiao, Y. Novel Small-Molecule Fluorophores for *In Vivo* NIR-IIa and NIR-IIb Imaging. *Chem. Commun. (Camb)* **2020**, *56* (22), 3289–3292.
